# Safeguarding Drosophila female germ cell identity depends on an H3K9me3 mini domain guided by a ZAD zinc finger protein

**DOI:** 10.1371/journal.pgen.1010568

**Published:** 2022-12-22

**Authors:** Laura Shapiro-Kulnane, Micah Selengut, Helen K. Salz

**Affiliations:** Department of Genetics and Genome Sciences, Case Western Reserve University School of Medicine, Cleveland, Ohio, United States of America; Geisel School of Medicine at Dartmouth, UNITED STATES

## Abstract

H3K9me3-based gene silencing is a conserved strategy for securing cell fate, but the mechanisms controlling lineage-specific installation of this epigenetic mark remain unclear. In *Drosophila*, H3K9 methylation plays an essential role in securing female germ cell fate by silencing lineage inappropriate *phf7* transcription. Thus, *phf7* regulation in the female germline provides a powerful system to dissect the molecular mechanism underlying H3K9me3 deposition onto protein coding genes. Here we used genetic studies to identify the essential cis-regulatory elements, finding that the sequences required for H3K9me3 deposition are conserved across *Drosophila* species. Transposable elements are also silenced by an H3K9me3-mediated mechanism. But our finding that *phf7* regulation does not require the dedicated piRNA pathway components, *piwi*, *aub*, *rhino*, *panx*, and *nxf2*, indicates that the mechanisms of H3K9me3 recruitment are distinct. Lastly, we discovered that an uncharacterized member of the zinc finger associated domain (ZAD) containing C2H2 zinc finger protein family, IDENTITY CRISIS (IDC; CG4936), is necessary for H3K9me3 deposition onto *phf7*. Loss of *idc* in germ cells interferes with *phf7* transcriptional regulation and H3K9me3 deposition, resulting in ectopic PHF7 protein expression. IDC’s role is likely to be direct, as it localizes to a conserved domain within the *phf7* gene. Collectively, our findings support a model in which IDC guides sequence-specific establishment of an H3K9me3 mini domain, thereby preventing accidental female-to-male programming.

## Author summary

Tissue development and function relies on cells remembering their identity. A cell’s identity is defined by the genes it expresses and those it does not. Recent work has shown that genes can be silenced by trimethylation of histone H3 lysine 9 (H3K9me3) marked chromatin, and that H3K9me3-mediated gene silencing is a vital strategy for securing cell fate. But there is very little information about how the machinery responsible for H3K9 methylation finds its target genes. Here we explore this issue using the *Drosophila* female germline where a mini domain of repressive H3K9me3 chromatin secures female germ cell fate by silencing *phf7*, a gene normally expressed in male germ cells. Transposable elements are also silenced by H3K9me3 mini domains, but we find that the proteins involved in this process are not required for *phf7* silencing. Instead, we find that silencing requires a previously uncharacterized protein, we have named IDENTITY CRISIS. Our work provides evidence that IDENTITY CRISIS directs the H3K9 methylation machinery to build a mini domain at the *phf7* locus. Our results shed new light into how cells safeguard their identity by silencing cell type inappropriate genes, and more specifically how these genes are identified by the silencing machinery.

## Introduction

Gene silencing is critical to establishing and maintaining cell fates. Once made, the decision to silence a gene is fortified by the acquisition of repressive histone modifications. While cell type specific epigenetic silencing is primarily associated with tri-methylation of histone H3 lysine 27 (H3K27me3)-marked chromatin, tissue specific genes can also be repressed by H3K9 methylation [1–4]. For example, in *S*. *pombe*, discrete H3K9me3 domains silence meiotic genes in vegetative cells (e.g., [5,6]). In *C*. *elegans*, H3K9 methylation silences inappropriate cell type specific genes, including germline genes, in somatic cells (e.g., [7,8]). In the *D*. *melanogaster* female germline, H3K9me3 silences male germline genes [9]. In the mouse, H3K9me3 silences germline genes during early embryonic development (e.g., [10,11]). Studies carried out in mammalian tissue culture systems further identify H3K9me3-mediated gene silencing as a conserved and vital strategy for maintaining cell fates in a wide range of tissues (e.g., [12–23]). However, the molecular mechanisms controlling tissue specific installation of this epigenetic modification onto protein-coding genes are largely unknown.

The repressive H3K9me3 histone modification has well characterized roles in constitutive heterochromatin formation, and the transcriptional silencing of repetitive DNA elements such as satellite repeats and transposable elements (TEs) [24–26]. These studies identified two mechanisms of H3K9me3 recruitment. One mechanism involves small RNAs that guide localization through a complementary base pairing mechanism. In *Drosophila* and mammals, for example, the PIWI-associated small RNAs (piRNAs) guide the H3K9me3 silencing machinery to TEs [27]. A second mechanism involves sequence specific DNA binding proteins. In mammals, for example, H3K9me3 can be guided to TEs by members of the vertebrate specific Kruppel-associated box zinc finger (KRAB-ZNF) family of DNA binding proteins [28–31]. An analogous mechanism might exist in *Drosophila*, as two zinc finger proteins, KIPFERL and SMALL OVARY, were recently shown to have roles in TE silencing [32–35]. Whether installation of this epigenetic modification onto protein-coding genes employs similar mechanisms remains unclear.

**Fig 1:**
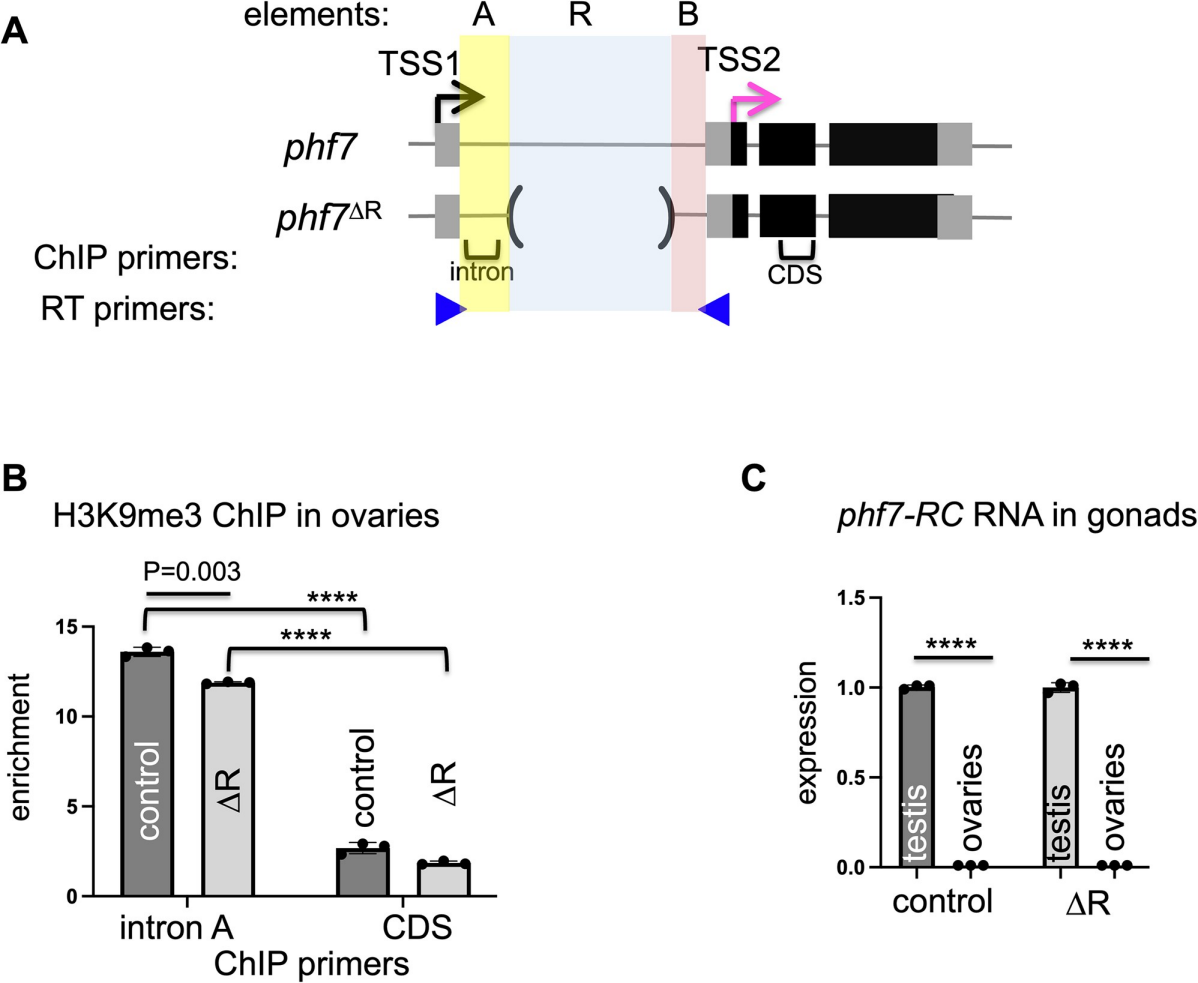
H3K9me3 deposition and sex specific transcriptional regulation are maintained in *phf7*^*ΔR*^ ovaries. **(**A) Diagram of the *phf7*^*+*^ and *phf7*^*ΔR*^ alleles. Exons are represented by boxes, flanking DNA, and introns by lines, black boxes are coding sequences, and grey boxes are untranslated regions (UTRs). The sequence elements A, R, and B location within the first intron are shaded in yellow, blue, and pink. In ovaries, transcription initiates from TSS2 (pink arrow), whereas an H3K9me3-mediated mechanism represses transcription from TSS1 (black arrow). Locations of primers for ChiP-qPCR and RT-qPCR are indicated by brackets & blue arrowheads. **(B)** H3K9me3 accumulation at the endogenous *phf7* locus in control (*y*^*1*^ *w*^*1*^*)* and mutant ovaries. ChIP-qPCR measured H3K9me3 signal at the *phf7* locus. ChIP to input enrichment is normalized to *rp49*. **(C)** Expression of the testis specific *phf7* transcript (*phf7-RC)* in control (*y*^*1*^ *w*^*1*^*)* and mutant ovaries. Expression is measured by RT-qPCR and is shown as the fold change relative to the testis. Expression is normalized to the total level of *phf7*. For both RT-qPCR and ChIP-qPCR experiments. Error bars represent the standard error of the mean (SD) of three biological replicates. A two-tailed Student’s t-test estimates statistical significance where ****p<0.0001.

In *Drosophila*, H3K9 methylation plays an essential role in securing female germ cell fate by silencing lineage inappropriate *PH**D* *f**inger protein* *7* (*phf7*) transcription [9,36]. Thus, *phf7* regulation in the female germline provides a powerful system to investigate how the H3K9me3 silencing mark is installed at protein-coding genes. *phf7* encodes a predicted chromatin reader, first identified in a screen for genes expressed in male but not female embryonic germ cells [37]. Curiously, *phf7* is not essential for male fertility, but it is critical that female germ cells not express the PHF7 protein. Forced expression of PHF7 activates a toxic gene expression program enriched for genes usually restricted to the male germline [36]. Even though PHF7 protein is limited to male germ cells, *phf7* is transcribed in both male and female germ cells. Sex specificity is achieved by alternative transcription start sites (TSS; **Fig 1A**). In testes, transcription from the upstream TSS (TSS1) produces a long mRNA isoform that makes protein. In ovaries, an H3K9me3 mini domain prevents the selection of TSS1. Instead, transcription initiates from the downstream TSS (TSS2) to produce a short mRNA isoform that is not translated and has no function. Of the three *Drosophila* enzymes known to methylate H3K9, only SETDB1 has a specific and nonredundant role in repressing *phf7* [9]. Germ cell specific loss of SETDB1, its binding partner ATF7IP, or the H3K9 reader HP1a produced ovarian germ cell tumors that inappropriately express the PHF7 protein. Notably, derepression results from losing the H3K9me3 mini domain. While these results establish the H3K9me3 mini domain controls *phf7* transcription, the mechanisms controlling installation of this epigenetic modification remains unclear.

We address the mechanism of targeted H3K9me3 deposition onto *phf7* by identifying essential cis-regulatory elements and trans-acting factors. We establish that the required cis-regulatory sequences are conserved across *Drosophila* species and find that the mechanism governing *phf7* regulation is different from what has been described for piRNA-guided H3K9me3 deposition on TEs. Lastly, we discover that repression depends on the previously unknown gene, *CG4936*, that we have named *identity crisis (idc)*. *idc* encodes a zinc finger associated domain (ZAD) containing C2H2 zinc finger protein. Notably, loss of *idc* in germ cells interferes with *phf7* repression by reducing H3K9me3 deposition. Together with the observation that IDC localizes to a conserved region within the *phf7* gene, our analysis supports a model in which IDC guides H3K9me3 installation, thereby preventing accidental female-to-male programming.

## Results

### Identification of cis-regulatory elements required for H3K9me3 deposition

H3K9me3 accumulates over a three kb region within the *phf7* gene [9]. This discrete peak covers the male TSS1, the first male-specific, non-coding exon, and most of the first intron. Interestingly, the intron contains seven copies of ~250 bp sequence not found anywhere else in the genome (element R; **Figs 1A and S1**). Repetitive elements can regulate gene expression in *cis* by serving directly as H3K9me3 nucleation sites or in *trans* by encoding small RNAs that serve as guides via a base pairing mechanism. We, therefore, hypothesized that the repeats play a role in H3K9me3 deposition and silencing of TSS1. To test this idea, we used CRISPR editing to produce animals deleted for the repeats in the endogenous locus (*phf7*^*ΔR*^*)* (**Fig 1A**). We were surprised to discover that homozygous *phf7*^*ΔR*^ animals are viable and fertile, suggesting that the repeats may not be essential for repression. To evaluate the impact of deleting the repeats in more detail, we measured H3K9me3 accumulation in wild-type and *phf7*^*ΔR*^ homozygous ovaries using chromatin immunoprecipitation followed by quantitative PCR (ChIP-qPCR). In wild-type (control) ovaries, sequences in the intron positioned 319 bp downstream of TSS1 (within intronic element A) are significantly enriched in the H3K9me3 modification when compared to the region within the coding sequence (CDS; *p*<0.0001) (**Fig 1B**). Significant H3K9me3 enrichment within element A compared to the CDS region (*p*<0.0001) was also observed in *phf7*^*ΔR*^ homozygous ovaries. Interestingly, there was less H3K9me3 accumulation within element A in *phf7*^*ΔR*^ than in controls (*p* = 0.003), raising the possibility that sex specific transcriptional regulation might be disrupted. In testes, transcription from the upstream TSS (TSS1) produces a long mRNA isoform, called *phf7-RC*. We therefore used RT-qPCR to assay for testis specific *phf7-RC* expression levels in wild-type and *phf7*^*ΔR*^ gonads. Contrary to our expectations, we found that *phf7*^*ΔR*^ did not disrupt sex specific transcription: As in wild-type gonads, *phf7-RC* expression was detected in *phf7*^*ΔR*^ homozygous testis but not ovaries (**Fig 1C**). These results indicate that the repeats are not essential for H3K9me3-mediated repression.

**Fig 2:**
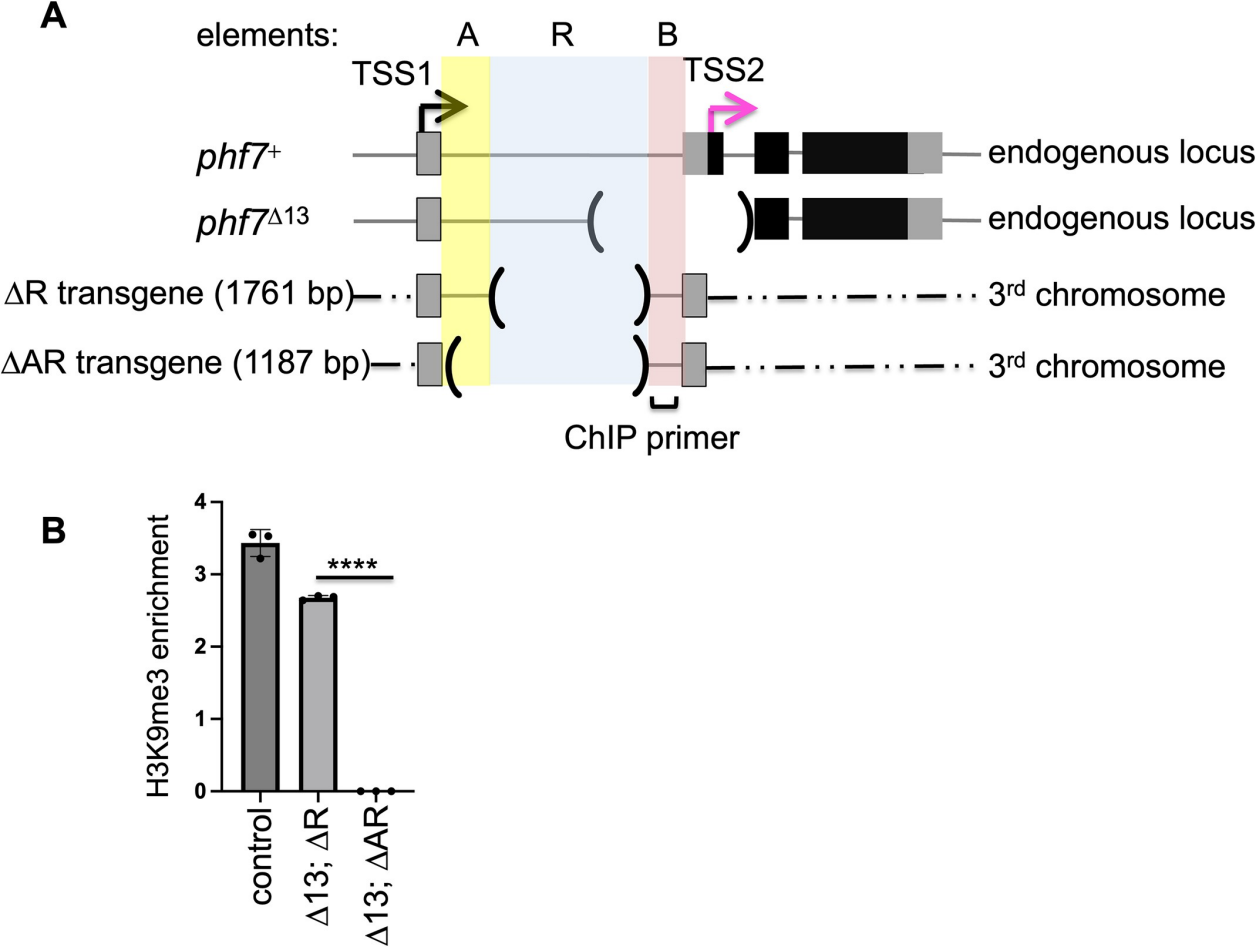
Non-coding sequences within the first intron are required for H3K9me3 deposition. **(A)** Diagram of *phf7*^*+*^, *phf7*^*Δ13*^, and the ΔR and ΔAR chromatin transgenic reporter lines inserted into the same 3^rd^ chromosomal site. **(B)** H3K9me3 accumulation on the transgenes. ChIP-qPCR measured signal, and the ChIP to input enrichment, normalized to *rp49*. Error bars represent the standard error of the mean (SD) of three biological replicates. A two-tailed Student’s t-test estimates statistical significance where ****p<0.0001.

To complement these studies, we used a transgenic approach to identify the sequences capable of promoting H3K9me3 when inserted into a heterologous genomic location on the 3^rd^ chromosome using site specific integration. We first created a transgene, that mimics the 5’ end of the *phf7*^*ΔR*^ mutant allele, extending from the first male specific exon to the beginning of the open reading frame in exon 2 (**S2 Fig**).To assay for H3K9me3 accumulation on the ΔR transgene, but not at the endogenous locus, we crossed each line into a *phf7*^*Δ13*^ background. *phf7*^*Δ13*^ is a 1.81 kb intragenic deletion allele, which allows us to measure the level of H3K9me3 deposition by ChIP-qPCR at a region that is present in the transgenes but absent in *phf7*^*Δ13*^ (intronic element B, **Fig 2A**). As expected, we found that these sequences included in the ΔR transgene promotes H3K9me3 accumulation. (**Fig 2A and 2B**). These data reinforce our conclusion that the tandem repeats (element R) are not required for H3K9me3 accumulation. In contrast, we found that H3K9me3 did not accumulate on a second transgene in which both elements A and R were removed (**Fig 2A and 2B**). We therefore conclude that the sequences that remain, including the first exon and region B, are not sufficient for H3K9me3 recruitment. These results also establish that element A contains cis-regulatory determinants required for H3K9me3 recruitment. It remains to be determined whether the element A determinants are sufficient for H3K9me3 recruitment or function redundantly with sequences within element R.

### Conservation of non-coding cis-regulatory elements

**Fig 3:**
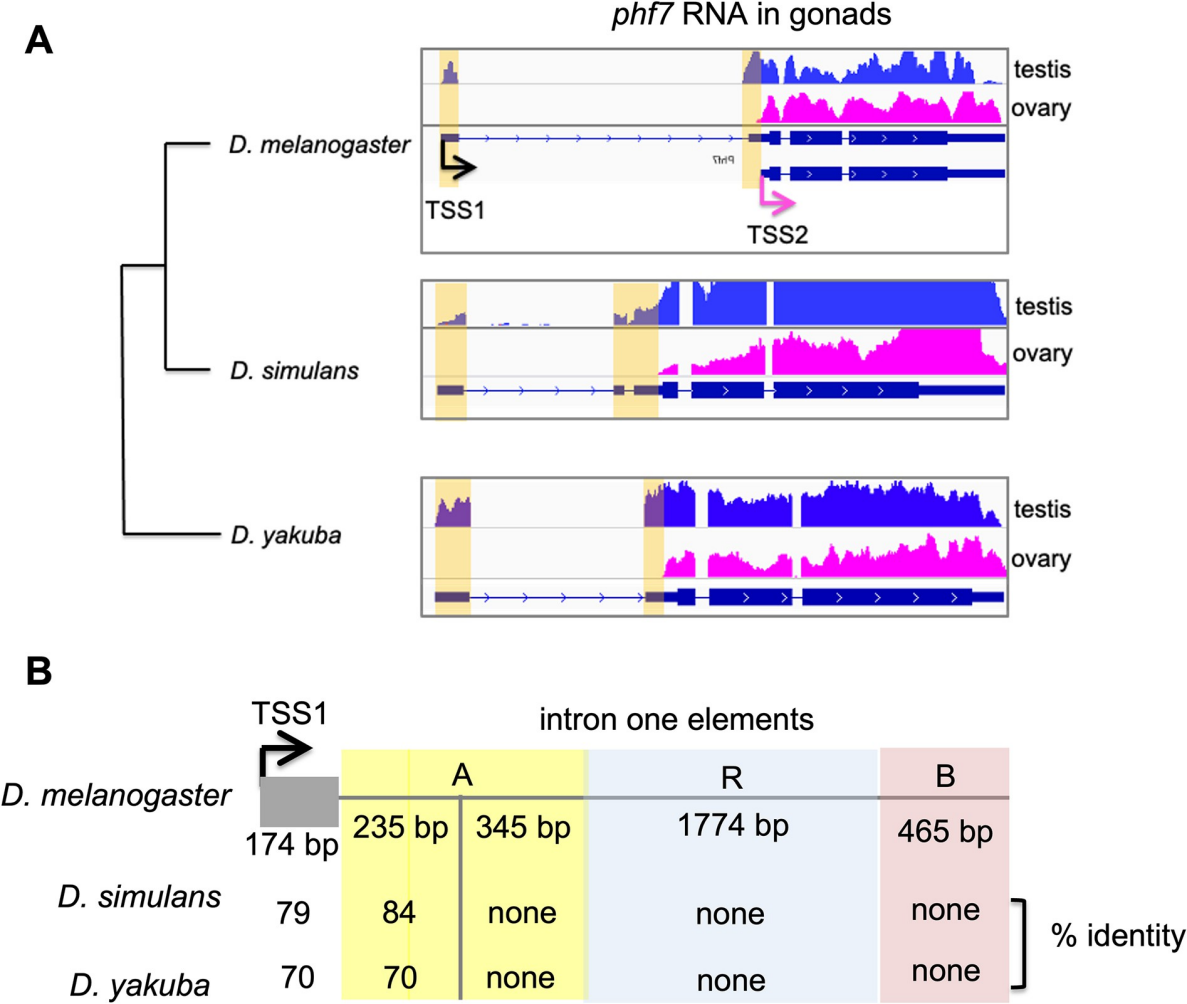
Sex specific transcriptional regulation is conserved. **(A)** Genome browser views of RNA-seq data aligned to the *phf7* locus from *D*. *melanogaster*, *D*. *simulans*, and *D*. *yakuba* ovaries and testis. **(B)** The first exon and a portion of the first intron are conserved. Pairwise alignments between the first exon and the adjacent intron were identified using the Basic Local Alignment Search Tool (BLASTn) available at (https://blast.ncbi.nlm.nih.gov). Percent identity to the Drosophila melanogaster to the first male specific exon and element A is shown. The sequence alignments are presented in S3 Fig.

Cis-regulatory elements are often conserved. Thus, we might expect the essential sequences within element A to be retained in evolution. To test this prediction, we chose to examine the level of conservation between *D*. *melanogaster*, *D*. *simulans*, and *D*. *yakuba*. Although *D*. *simulans* and *D*. *yakuba* are separated from *D*. *melanogaster* by 5–15 million years, the *phf7* intron/exon structure is conserved (**Fig 3A**). Furthermore, RNA-seq data obtained from wild-type testis and ovaries [38,39] shows that *phf7* expression is sex specific in *D*. *simulans* and *D*. *yakuba* (**Fig 3A)**. Together these observations suggest that sex-specific transcriptional regulation through alternative TSS selection is conserved between *D*. *melanogaster*, *D*. *simulans*, and *D*. *yakuba*. When we compared the level of sequence conservation in the first intron between *D*. *melanogaster*, *D*. *simulans*, and *D*. *yakuba*, we found that only the first 235 bp of intron element A was conserved (**Figs 3B and S3**). Interestingly, the first non-coding 174 bp exon also displays a high level of conservation, suggesting that additional cis-regulatory elements may be present within the first non-coding exon (**Figs 3B and S3**)

### Core components of the piRNA pathway are not required for *phf7* sex specific transcriptional control

**Fig 4:**
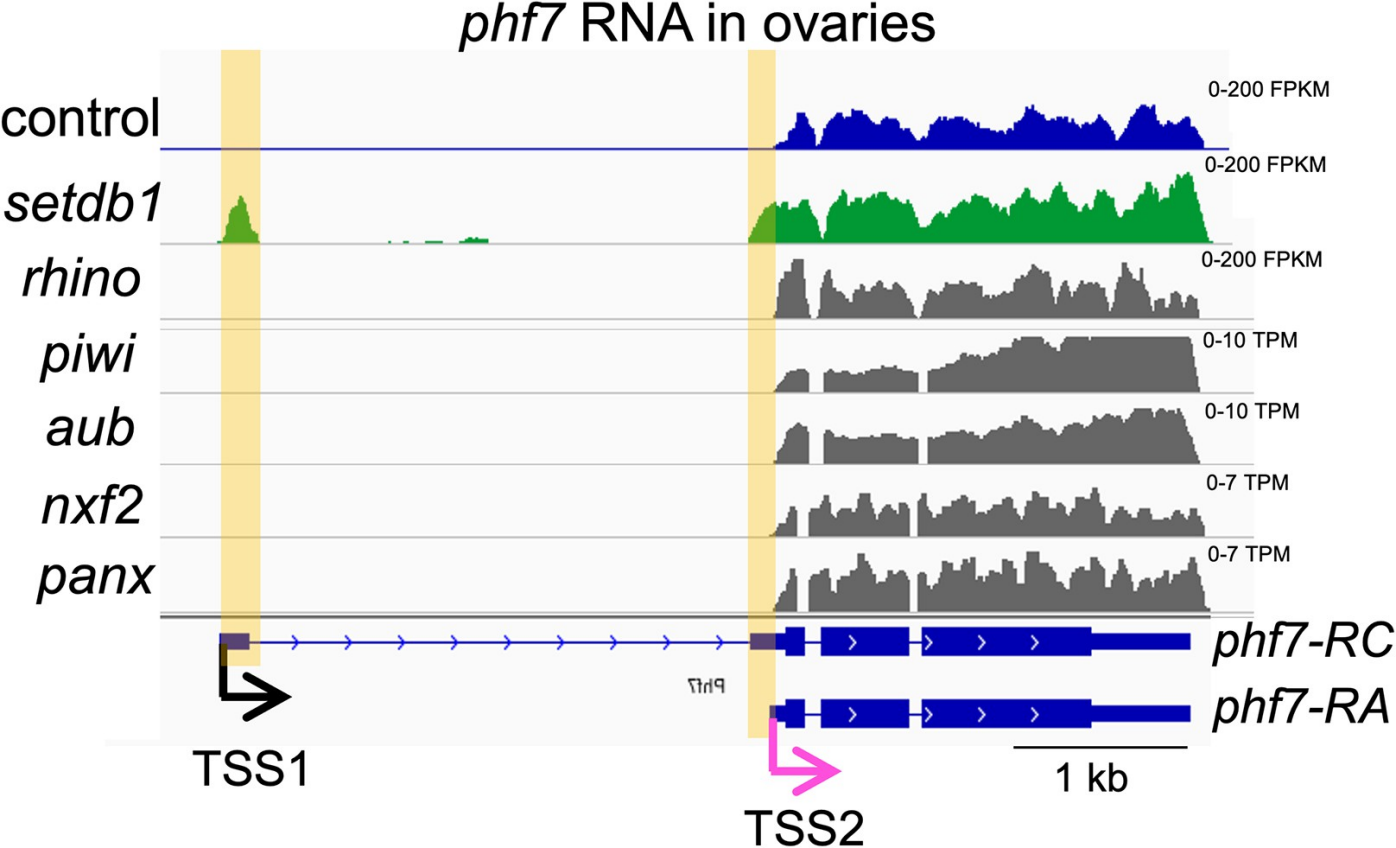
Depletion of *setdb1*, but not the dedicated piRNA-pathway components, leads to female-to-male reprogramming at the *phf7* locus. Genome browser views of the *phf7* locus. Tracks show RNA-seq reads from control and mutant ovaries aligned to the Drosophila genome (UCSC dm6). The screenshot is reversed so that the 5’ end of the gene is on the left. The RNA-seq reads that are unique to *setdb1* mutant ovaries are highlighted. In wild-type control ovaries, only the *phf7-*RA transcript is detected (TSS2, pink arrow), whereas loss of *setdb1* leads to ectopic expression of the testis specific *phf7-RC* isoform (TSS1, black arrow). In contrast, mutations in the *rhino*, *piwi*, *aub*, *nxf2* and *panx* genes do not disrupt sex specific *phf7* transcription. The complete mutant genotypes and accession numbers of the RNA-seq data sets are listed in S3 Table.

Prior studies have shown that SETDB1 is required for both TE and *phf7* silencing in germ cells [9,40–42]. Although there are no recognizable TE sequences at *phf7*, the piRNA pathway may nevertheless play a role in repressing *phf7*. To test this possibility, we analyzed published RNA-seq data sets from ovaries carrying mutations in genes encoding the dedicated piRNA pathway components, *piwi*, *aubergine (aub)*, *rhino*, *panoramix (panx*, also known as *silenceo)*, and *nuclear RNA export factor 2 (nxf2)* [43–45]. Loss of each component causes the piRNA pathway to collapse. *piwi*, *aub* and *rhino* are essential for piRNA biogenesis [42, 43,46]. *panx* and *nxf2*, while dispensable for piRNA biogenesis, are necessary for SETDB1 to deposit H3K9me3 marks onto TEs [45, 47–51]. In agreement with our prior studies [9], only the shorter *phf7-RA* transcript is detectable in wild-type ovaries, but the longer testis specific *phf7-RC* transcript is present in *setdb1* mutant ovaries **(Fig 4)**. In contrast to the *setdb1* mutant ovaries, *piwi*, *aub*, *rhino*, *panx*, and *nxf2* mutant ovaries only express the shorter *phf7-RA* transcript, indicating that transcriptional regulation is not disrupted. Based on these findings we conclude that the mechanisms controlling H3K9me3 deposition onto *phf7* and TEs are distinct.

### The ZAD-ZNF protein IDC is required for *phf7* sex specific transcriptional control

In mammalian cells, SETDB1 can be recruited to its targets by members of the large KRAB-zinc finger family of sequence specific DNA binding proteins [28–30]. The KRAB family is confined to mammals. It has been hypothesized that members of the insect specific ZAD-ZNF family might be functional analogues [52–54]. We recently tested the function of 68 of the 93 ZAD-ZNF encoding genes in female germ cells by performing an RNAi screen [55]. This screen identified eight ZAD-ZNF genes required for oogenesis. Here, we focus on CG4936 which we name *identity crisis* (*idc*). *idc* encodes a 521 amino acid (aa) protein that contains an N-terminal ZAD (zinc finger associated domain, 22–95 aa), an unstructured linker region, and a C-terminal domain that includes an array of 5 C2H2 zinc fingers (386–491 aa). Studies of other ZAD-ZNF proteins suggest that the ZAD mediates protein-protein interactions and the C2H2 zinc fingers bind DNA [56–60]. Interestingly, the knockdown phenotype of IDC suggests a defect prior to oocyte specification [55]. Because this phenotype is reminiscent of the germ cell defects caused by ectopic PHF7 expression, we hypothesize that IDC is required for *phf7* repression.

**Fig 5:**
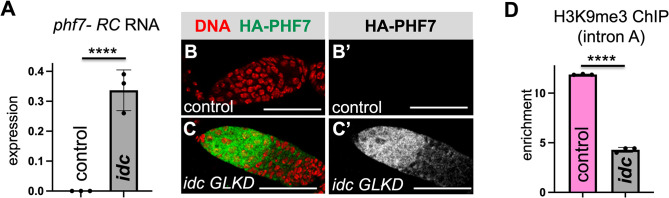
*idc* germ cell specific knock-down disrupts *phf7* regulation. **(A)** *idc* GLKD germ cells (*nos>idc-RNAi*) express the testis *phf7-RC* transcript. RT-qPCR measures expression normalized to the total level of *phf7* in RNA extracted from control *(nos>white-RNAi*) and *idc* GLKD (*nos>idc-RNAi*) ovaries. The histograms show the mean ± SD of three biological replicates. **(B, C)** *idc* GLKD germ cells inappropriately express the PHF7 protein. Confocal images of ovaries dissected from control (*nos>white-RNAi*) and mutant (*nos>idc-RNAi*) females carrying an HA-PHF7 transgene and stained for HA (green, white in B’ and C’) and DNA (red). Scale bar 50μm. **(D)** H3K9me3 accumulation at the endogenous *phf7* locus is reduced in *idc* GLKD ovaries. ChIP-qPCR measured H3K9me3 signal at the *phf7* locus (primer in region A) in control (*nos>white-RNAi)* and mutant (*nos>idc-RNAi*) ovaries. ChIP to input enrichment is normalized to *rp49*. The histogram shows the mean ± SD of three biological replicates. A two-tailed Student’s t-test estimates statistical significance where ****p<0.0001.

To investigate the possibility that loss of *idc* in female germ cells disrupts *phf7* repression, we first used RT-qPCR to assay for the presence of the testis specific *phf7-RC* transcript. Germ cell specific knock down (GLKD) was achieved by expressing a germline optimized inducible RNA interference (RNAi) transgene with the germ cell specific *nos-GAL4* driver. We demonstrated knockdown efficiency by showing that *idc GLKD* significantly reduces IDC protein levels in female germ cells, but not in the surrounding somatic cells (**S4 Fig**). To rule out indirect effects arising from RNAi knockdown, we also knocked down *white*, which is not expressed in germ cells, as a negative control. Using primer pairs capable of detecting *phf7-RC*, we found that in contrast to control ovaries, *phf7-RC* is detectable in *idc* GLKD mutant ovaries (**Fig 5A**). We then asked whether ectopic *phf7-RC* expression correlated with ectopic PHF7 protein expression. Previous work has shown that a BAC transgene that encodes an N-terminally HA-tagged PHF7 protein (HA-PHF7) is a faithful reporter of the sex specific protein expression pattern of PHF7 [37,61]. We found that, in contrast to wild-type ovaries, the HA-PHF7 protein is detectable in the cytoplasm and the nucleus of the *idc GLKD* mutant ovaries (**Fig 5B and 5C**). We conclude that IDC is required for *phf7* regulation.

To test whether *idc* plays a role in controlling H3K9me3 deposition, we compared the amount of H3K9me3 accumulation within element A in wild-type and mutant ovaries using ChIP-qPCR. We found that H3K9me3 was significantly reduced in *idc* GLKD ovaries compared to control ovaries (*p*<0.0001; **Fig 5D**). Based on these studies, we conclude that IDC regulates *phf7* transcription by promoting H3K9me3 deposition.

### IDC is a nuclear protein that associates with DNA in ovaries

**Fig 6:**
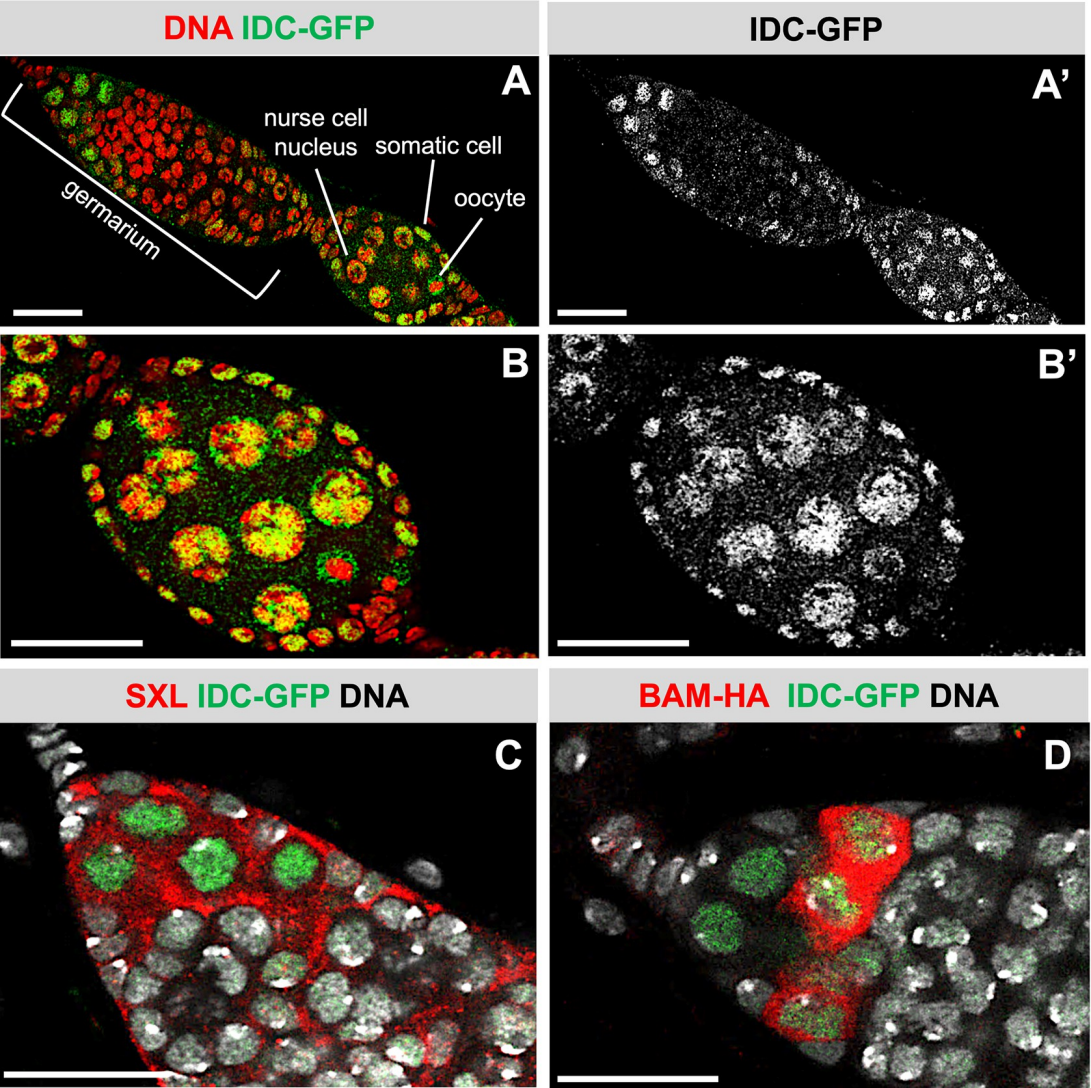
IDC is nuclear protein expressed in both somatic and germline cells. **(A)** Confocal image of an ovariole dissected from a female carrying the IDC-GFP rescue transgene stained for GFP (green, white in A’) and DNA (red). The bracket indicates the germarium located at the anterior end of the ovariole. Scale bar 25μm. **(B)** Confocal image of an early-stage egg chamber dissected from a female carrying the IDC-GFP transgene stained for GFP (green, white in B’) and DNA (red). Scale bar 25μm. **(C)** Confocal image of the anterior end of a germarium dissected from a female expressing the IDC-GFP fusion protein and co-stained for GFP (green), DNA (white), and SXL (red). Scale bar 25 μm. **(D)** Confocal image of the anterior end of a germarium dissected from a female expressing the IDC-GFP and the BAM-HA fusion proteins co-stained for GFP (green), DNA (white), and HA (red). Scale bar 25μm.

Published modENCODE RNA-seq data sets indicates that the *idc* RNA is broadly expressed throughout development [62]. To examine IDC protein expression in the ovary, we used a genomic fosmid transgene that encodes a C-terminally GFP-tagged IDC protein (IDC-GFP). We inferred that the GFP tag does not interfere with *idc* function because the *idc-GFP* rescues the *idc*^*1*^ lethal phenotype (see Materials and Methods). Each ovary is comprised of 16–20 ovarioles. Each ovariole contains germ cells spanning the range of maturity from germline stem cells (GSCs) at the anterior end to fully mature eggs at the posterior end [63]. We observed IDC-GFP in the somatic cells, the nurse cells and the oocyte (**Fig 6A**). Notably, the IDC-GFP protein is nuclear. Furthermore, IDC-GFP is tightly associated with the nurse cell polytene chromosomes, consistent with its presumed DNA binding activity (**Fig 6B**).

In the germarium, at the anterior end of the ovariole, we observed prominent IDC-GFP staining in the nucleus of only 3 to 6 germ cells (**Fig 6A)**. This expression pattern is like that described for the female specific sex determination protein SEX-LETHAL (SXL), which is also required for *phf7* H3K9me3 silencing [9, 64,65]. Indeed, co-staining experiments reveal that SXL accumulates in the cytoplasm of all IDC-GFP expressing germ cells (**Fig 6C**). Studies have shown that SXL accumulates in GSCs and their immediate daughter cells. When GSCs divide, the daughter cells at the tip remain a GSC, while the more posterior daughter cells, called a cystoblast (CB), express the BAG OF MARBLES protein (BAM). In agreement with published studies, BAM expression, assessed with a transgene that encodes a C-terminally HA-tagged BAM protein expressed from a *bam* promoter is readily detectable in the cytoplasm of just a few germ cells [66]. Co-staining experiments reveal that IDC-GFP is expressed in the GSCs where BAM-HA is not expressed as well as in the adjacent two to three cells where BAM-HA is detectable (**Fig 6D**). Together, these observations indicate that the nuclear IDC protein is broadly expressed, with prominent expression in the GSCs and their immediate daughter cells.

### IDC binds to sequences within the male specific exon

**Fig 7:**
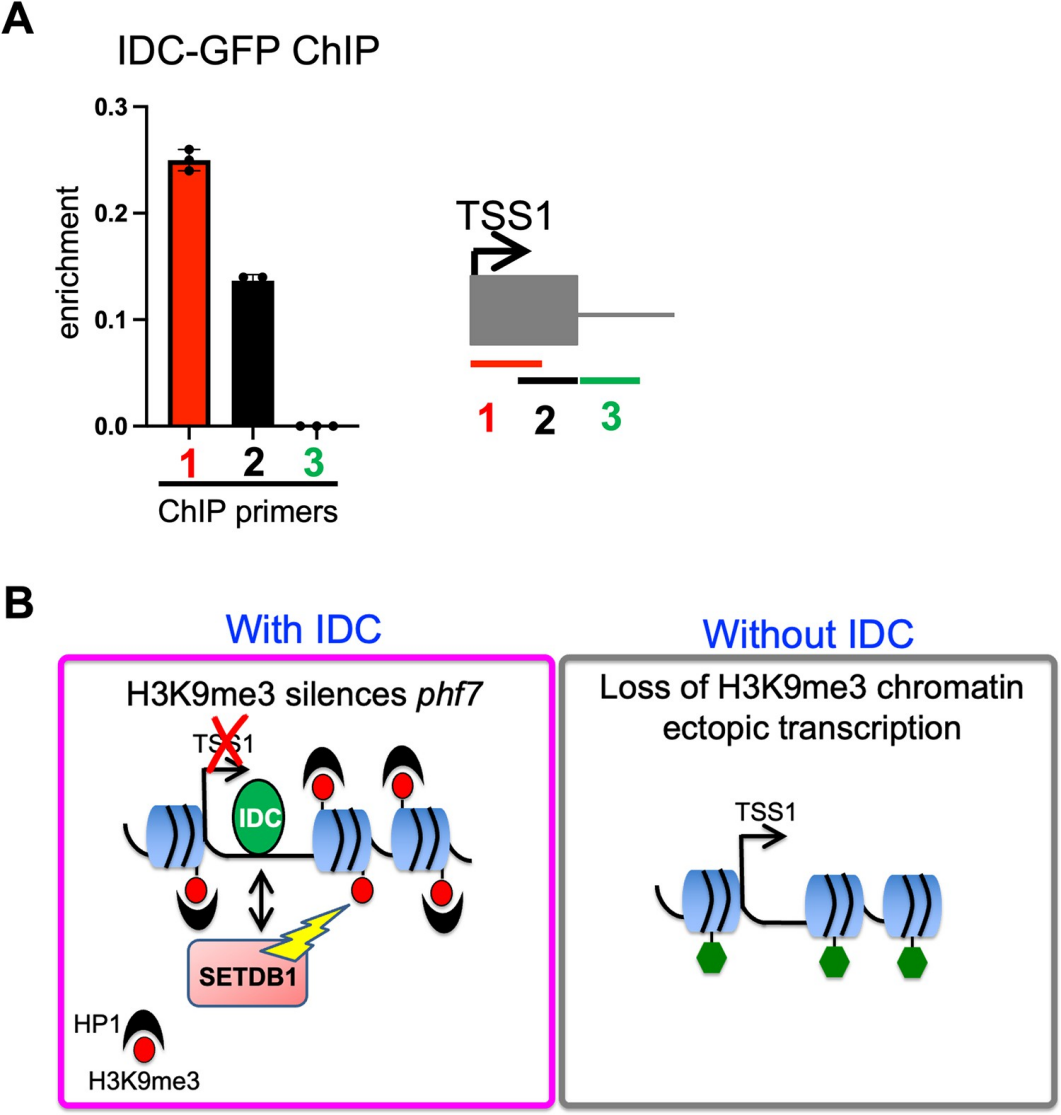
IDC binds to *phf7*. **(**A) ChIP-qPCR shows that IDC binds to the *phf7* first exon. Left: The histogram shows the mean ± SD of three biological replicates. Right: Cartoon showing the location of the primers used for ChIP-qPCR. **(B)** Model for H3K9me3-mediated silencing of *phf7*. In female germ cells, IDC binds to *phf7*, directing H3K9me3 deposition by SETDB1 H3K9 methytransferase. HP1 binds to H3K9me3, resulting in transcriptional silencing. In germ cells lacking IDC, the dissolution of the H3K9me3 domain correlates with ectopic testis-specific *phf7-RC* transcription and PHF7 protein expression. Ectopic PHF7 activates a toxic gene expression program enriched for genes usually restricted to the male germline.

IDC contains five zinc fingers suggesting that it has sequence specific DNA binding activity. We speculated that IDC might promote H3K9me3 deposition by binding to the *phf7* locus. To test this prediction by ChIP-qPCR, we designed primers along the length of the conserved noncoding sequences in the first exon and the intron. These experiments revealed that the IDC-GFP tagged protein associates with chromatin within the first non-coding exon, but not outside of it (**Fig 7A**). Collectively, our studies indicate that IDC regulates *phf7* directly by serving as an H3K9me3 guidance factor.

## Discussion

SETDB1-controlled H3K9 methylation plays an essential role in securing female germ cell fate by silencing lineage-inappropriate *phf7* transcription [9,36]. SETDB1 is also required for TE silencing, where a piRNA-guided mechanism guides H3K9me3 deposition [40–42]. In this work, we establish that *phf7* is silenced by a piRNA-independent mechanism and discover that regulation depends on IDC, an uncharacterized member of the ZAD-ZNF family of DNA binding proteins. Regulation appears direct, as IDC is required for H3K9me3 deposition and localizes to the conserved first exon of *phf7* in ovarian extracts. Collectively, our data establish that the sequence specific DNA binding protein IDC directs the H3K9 methylation machinery to build a silencing domain at the *phf7* locus, thereby preventing accidental female-to-male reprogramming **(Fig 7B)**. In addition to extending our understanding of how female germ cell fate is maintained, our studies provide the first example of a ZAD-ZNF protein guiding H3K9me3-mediated gene silencing.

Although our work is consistent with a simple model in which the SETDB1 H3K9me3 methyltransferase is recruited to *phf7* by IDC, the mechanism by which IDC guides the methylation machinery to *phf7* remains an open question. For example, it remains unclear whether recruitment is direct, as our attempts to co-immunoprecipitate SETDB1 and IDC were unsuccessful. Furthermore, while we establish that IDC is required for H3K9me3 recruitment, our chromatin transgenic reporter assays show that the region to which it binds, the conserved first exon, is not sufficient. This observation, together with our identification of a second conserved cis-regulatory element within the adjoining intron invites speculation that IDC works in conjunction with other sequence-specific recruitment factors. One attractive contender is *stonewall (stwl)*. STWL is a heterochromatin-associated protein that acts as a transcriptional repressor *in vitro* [67], associates with SETDB1 in yeast [68] and localizes to the *phf7* locus in S2 cells [69]. Importantly, loss of *stwl* in ovaries leads to the inappropriate expression of the testis *phf7* transcript [69]. Future studies focused on the rules that govern female-specificity may reveal a general mechanism for context-dependent establishment of H3K9me3 silencing domains.

We have previously established that the H3K9me3 reader protein, HP1a, is essential for *phf7* silencing [9]. A requirement for HP1a is not surprising, as HP1a drives chromatin compaction and transcriptional silencing [70]. HP1a also recruits H3K9 methyltransferases, enabling the spreading of the H3K9me3 repressive domain through a positive feedback mechanism. The spread of H3K9me3 over the three kb region within the *phf7* gene likely occurs by an analogous mechanism. At the *phf7* locus, however, the H3K9me3 domain does not extend into the open reading frame or the neighboring genes. Therefore, more work is needed to understand what stops the spreading of this repressive chromatin domain.

The SETDB1-controlled H3K9 methylation silencing pathway in female germ cells is not restricted to *phf7*. Genome-wide H3K9me3 profiling in wild-type and mutant ovaries has shown that SETDB1 silences two classes of protein-coding genes. One type includes genes usually expressed in the testis [9]. A second class includes genes that are typically expressed in undifferentiated female germ cells but are silenced once the oocyte is specified, such as *ribosomal protein S19b* [71]. These few examples of context dependent H3K9me3 gene silencing suggest a finely tuned guidance mechanism in the female germline. It will be interesting to explore whether other members of the ZAD-ZNF gene family serve as H3K9me3 guidance factors.

In summary, by focusing on a single biologically relevant gene, we discovered a putative DNA binding protein that guides the installation of a H3K9me3 repressive domain onto a protein-coding gene. These findings are reminiscent of how TEs can be silenced in mammals wherein members of the KRAB-ZNF protein family recruit SETDB1 to establish epigenetic repression [28–31]. Interestingly, the KRAB-ZNFs are vertebrate specific, and the ZAD-ZNFs are insect specific. Yet, both gene families exhibit similar patterns of species-specific gene expansion and diversification. These observations have fueled the speculation that ZAD-ZNFs and KRAB-ZNFs perform similar functions [52–54]. In fact, IDC’s closest human relative is ZNF75D, a KRAB-ZNF protein of unknown function. Whether this or other members of the KRAB-ZNF protein family are required for tissue specific H3K9me3 gene silencing will be an exciting area of further exploration.

## Materials and methods

### *Drosophila* stocks and culture conditions

Fly strains were maintained on standard molasses food at 25°C unless otherwise noted. Wild-type control flies were from the lab *y*^*1*^ *w*^*1*^ stock. Stocks used to report on protein gene activity include the HA-tagged *phf7* transgene, *PBac{3XHA-PHF7}* [37]; the GFP-tagged *idc* transgene, *FlyFos{fTRG00376*.*sfGFP-TVPTBF}* (VDRC #318556) described in [72] and the HA-tagged *bam* transgene, *P{Bam*::*HA}* [66]. *phf7*^*Δ13*^, *phf7*^*ΔR*^, the *idc*^*1*^ null allele, and the ΔR and ΔAR chromatin reporters were generated for this study, as described below.

Crosses for knock-down studies were set up at 29°C, and adults were aged 3–5 days before gonad dissection. Knock-down studies were carried out with the following germline-optimized lines generated by the *Drosophila* Transgenic RNAi Project [73]: *idc*-*P{TRiP*.*HMC05569}* (BDSC #64550), and the control *w-P{TRiP*.*GL00094}* (BDSC #35573). Although off-target effects remain a concern for RNAi-induced knockdown studies, neither RNAi line is predicted to have off-target activity (https://www.flyrnai.org/up-torr/UptorrFly.jsp). Expression of the UAS-RNAi transgenic lines was driven by the third chromosome *P{GAL4*::*VP16-nos*.*UTR}* insertion (BDSC #4937), which is strongly expressed in germ cells.

### Generation of the *phf7*^*ΔR*^ allele

We generated the *phf7*^*ΔR*^ allele in two steps. First, CRISPR was used to replace the repeats within the first intron with a 3XP3::DsRed cassette by Rainbow Transgenic Flies, Inc. The deletion was generated with the following guide RNAs: agttaaaaaaaatcaatcgatgg and cgcagcgattgaatgttaatggg. 1 kb homology arms were generated through PCR and cloned into the pHD-dsRed-attP (Addgene #51019) [74]. Guide RNAs and the donor vector were co-injected into *nos-Cas9* embryos by Rainbow Transgenic Flies. Flies were screened for DsRed expression in the eyes, and sequence verified for accuracy. *phf7*^*ΔR*^ was generated by removing the DsRed cassette. Homozygous *phf7*^*ΔR+dsRED*^ females were crossed to a Cre-expressing fly line (BDSC #1092), and the male progeny screened for the loss of dsRED expression in the eyes, followed by sequence confirmation of precise tag excision.

### Generation of the *phf7*^*Δ13*^ allele

The *phf7*^*Δ13*^ allele was generated by P-element mediated imprecise excision as follows: females homozygous for the P-element insertion P{EPgy2}Phf7^EY03023^ (BDSC #15894) were crossed with the Δ2–3 transposase expressing line (BDSC #3664). Potential excision lines were established from the male progeny of the F1 females that had lost the *white*^*+*^ marker carried by the P element. Stocks that carried deletions were identified by PCR. The exact breakpoints were determined by DNA sequencing the PCR amplified DNA fragments.

### Generation of the ΔR and ΔAR chromatin transgenic reporter lines

Constructs were generated by VectorBuilder’s custom cloning services (https://en.vectorbuilder.com). The *phf7* sequences, described in **S2 Fig,** were inserted into their “user-defined promoter” modification of the pUASTattB expression vector. The vectors were sent to Rainbow Transgenic Flies Inc. for *phi-C31* catalyzed integration into the 65B2 PBac{y[+]-attP-3B}VK00033 site. Transgenic flies were sequence verified for accuracy.

### Generation of the *idc*^*1*^ null allele and rescue by the GFP-tagged *idc* transgene

The *idc*^*1*^ allele was generated by *in vivo* CRISPR mutagenesis [75]. Females expressing two sgRNAs under control of the GAL4/UAS system, P{HD_CFD00598} (VDRC #341525), were crossed at 25°C to M{vas-Cas9}; P{GAL4::VP16-nos.UTR} (BDSC #55821 + #4937) males. The *vas-Cas9; nos>gRNA* male offspring were crossed to a third chromosome balancer line to isolate and balance each putative *idc* allele in the next generation. *idc* alleles were identified by the failure to complement Df(3R)ED6027 (BDSC #9479) and checked for the presence of the predicted indel by PCR.

We found that *idc*^*1*^*/Df(3R)ED6027* animals do not survive to adulthood (n>100). To verify that the GFP-tagged *idc* transgene, PBac{fTRG00376.sfGFP-TVPTBF} (VDRC #318556), rescues *idc*^*1*^, we first made the double mutant *idc*^*1*^, *idc-GFP*. To test for rescue, we crossed *idc*^*1*^, *idc-GFP/TM3* to *Df(3R)ED6027/TM3* males. This cross yielded 84% (31/37) of the expected *idc*^*1*^, *idc-GFP/ Df(3R)ED6027* progeny, all of which were fertile.

### qRT-PCR and data analysis

RNA was extracted from dissected gonads using TRIzol (Thermo Fisher, cat# 15596026). Quantity and quality were measured using a NanoDrop ND-1000 spectrophotometer. The RNA was treated with DNase RQ1 (Promega, cat# M6101). cDNA was generated by reverse transcription using a SuperScript First-Strand Synthesis System for RT-PCR kit (Thermo Fisher, cat# 11904018) using random hexamers. qPCR was performed using *Power* SYBR Green PCR Master Mix (Thermo Fisher, cat# 4368706) with an Applied Biosystems QuantStudio 3 Real-Time PCR system. PCR steps were as follows: 95°C for 10 minutes followed by 40 cycles of 95°C for 15 seconds and 60°C for 1 minute. Melt curves were generated with the following parameters: 95°C for 15 seconds, 60°C for 1 minute, 95°C for 15 seconds, and 60°C for 15 seconds. Measurements were taken in biological triplicate with two technical replicates each. Relative transcript levels were calculated using the 2-ΔΔCt method [76]. Using GraphPad Prism software, P values were calculated using unpaired two-tailed Student’s t-tests. The primers used for measuring RNA levels are listed in **S1 Table**.

### ChIP-qPCR and data analysis

ChIP was performed as described in [9] with some modifications. Buffers are listed in **S2 Table**. Briefly, ovaries from 200 1–2 day old adults were dissected in PBSP, homogenized with a pellet pestle, and crosslinked with 1.8% methanol-free formaldehyde (Thermo Fisher, cat# 28906) for 5 minutes. Fixation was quenched for 5 minutes by adding glycine to a final concentration of 225mM. The solution was removed, and the pellet was washed in PBSP 3 times. Samples were resuspended in 500μl PBSP and stored at -80°C. Samples were thawed, centrifuged, and resuspended in 1ml lysis buffer 1. Samples were homogenized using sterile homogenizing beads in a Bullet Blender Homogenizer at 4°C for six cycles of 30 seconds on and 1 minute off. Following centrifugation, cell pellets were washed at 4°C for 10 minutes in lysis buffer 1, centrifuged, washed for 10 min at 4°C in lysis buffer 2, centrifuged and then resuspended in 750μl lysis buffer 3. Chromatin was sheared to 100–500 base pairs at 4°C with a Diagenode BioRuptor Pico for 15 cycles of 30 seconds on, and 30 seconds off.

For H3K9me3 ChIP, the chromatin lysates were precleared with a 1:1 mix of protein A/G Dynabead magnetic beads (Thermo Fisher, cat# 10001D and 10003D) for 1 hour at 4°C and then incubated overnight at 4°C with 1:1 mix of protein A/G Dynabead magnetic beads conjugated to H3K9me3 antibody (Abcam, cat# 8898 RRID: AB_306848).

For GFP ChIP, the chromatin lysates were precleared with Chromotek Magnetic Binding Control Agarose Beads (Proteintech, cat# bmab) for 1 hour at 4°C, and then incubated overnight at 4°C with Chromotek GFP-Trap Magnetic Agarose beads (Proteintech, cat# gtma).

Following immunoprecipitation, the samples were washed six times with ChIP-RIPA buffer, two times with ChIP-RIPA/500 buffer, two times with ChIP-LiCl buffer, and twice with TE buffer. DNA was eluted and reverse crosslinked from beads in 200μl elution buffer overnight at 65°C. Following RNaseA and proteinase K treatment, DNA was recovered by phenol-chloroform extraction and used for qPCR.

qPCR was performed using *Power* SYBR Green PCR Master Mix (Thermo Fisher, cat# 4367659) with an Applied Biosystems QuantStudio 3 Real-Time PCR system. PCR steps were as follows: 95°C for 10 minutes followed by 40 cycles of 95°C for 15 seconds and 60°C for 1 minute. Melt curves were generated with the following parameters: 95°C for 15 seconds, 60°C for 1 minute, 95°C for 15 seconds, and 60°C for 15 seconds. Primers as listed in **S1 Table**. ChIP experiments were performed on three independent biological samples. qPCR measurements on each sample were performed on at least two technical replicates. For H3K9me3 ChIP-qPCR, the ChIP to input enrichment (presented as percent input) is normalized to a negative control genomic region in the *rp49* locus. Statistical analysis was carried out by the GraphPad Prism software. The P values were calculated using unpaired two-tailed Student’s t tests.

### Immunofluorescence and image analysis

Females were aged 3–5 days before gonad dissection. Ovaries were fixed and stained according to standard procedures with the following primary antibodies: mouse anti-*Drosophila* SXL (1:100, Developmental Studies Hybridoma Bank, cat# M18, RRID: AB_528464), rabbit anti-GFP (1:2500, Thermo Fisher, cat# A-11122, RRID: AB_221569) or FITC conjugated goat anti-GFP (1:750, Abcam, cat# ab6662, RRID: AB_305635), and rat anti-HA high affinity (1:500, Sigma, cat# 11867423001, RRID: AB_390919). The following secondary antibodies were used at 1:200: Alexa Fluor 555 goat anti-rat (Thermo Fisher, cat# A-21434, RRID: AB_2535855), FITC goat anti-mouse (Jackson Immunoresearch Laboratories, cat# 115-095-003, RRID: AB_2338589), or FITC goat anti-rabbit (Jackson Immunoresearch Laboratories, cat# 111-095-003, RRID: AB_2337972). TO-PRO-3 Iodide carbocyanine monomer nucleic acid stain (1:1000, Thermo Fisher, cat# T3605) was used to stain DNA. Images were taken on a Leica SP8 confocal with 1024x1024 pixel dimensions, a scan speed of 600 Hz, and a frame average of 3. Sequential scanning was done for each channel, and three Z-stacks were combined for each image. Processed images were compiled with Microsoft PowerPoint.

### Analysis of publicly available RNA-seq data sets

The sources of the publicly available RNA-seq data generated from wild-type and mutant *Drosophila* tissues are listed in **S3 Table**. The data were downloaded from the SRA database at NCBI and analyzed using the tools available on the Galaxy web platform (https://usegalaxy.org/). FastQC assessed read quality and STAR was used to align the reads to the *Drosophila* reference genome (UCSC dm6). Screenshots of the expression data displayed on The Integrated Genome Viewer (IGV) are shown.

*D*. *simulans* and *D*. *yakuba phf7* orthologs were identified using the ortholog database at www.flybase.org. RNA-seq data from wild-type *D*. *simulans* and *D*. *yakuba* gonads was downloaded from the SRA database at NCBI (**S4 Table**). We uploaded the sequencing data to the Galaxy web platform (https://usegalaxy.org/), assessed read quality with FastQC and used STAR to align the reads to their respective reference genomes (listed in **S4 Table**). Browser screenshots of the expression data are displayed on the Integrated Genome Viewer (IGV).

### DNA sequence alignments

Multiple sequence alignments were performed in SnapGene (https://www.snapgene.com) using MUSCLE with default options. Pairwise sequence alignments were performed in BLASTn (https://blast.ncbi.nlm.nih.gov).

## Supporting information

S1 Fig The first intron of *phf7* contains seven copies of an ~250 bp DNA sequence. Sequence alignment of the 7 ~250 bp sequences generated with the multiple sequence alignment tool MUSCLE embedded in the SnapGene software (snapgene.com).
(PDF)

S2 Fig Generation of the ΔR and ΔAR chromatin transgenic reporter lines. ΔR transgene was designed to mimic the 5’ end of the *phf7*^*ΔR*^ mutant gene, extending from the first male specific exon to the beginning of the open reading frame in exon 2. In the sequence below, the deleted R element is highlighted in purple. The ΔAR transgene contains a 2^nd^ deletion, highlighted in yellow (region A). The sequences highlighted in blue and the primers located in region B). The constructs were generated by VectorBuilder’s custom cloning services (https://en.vectorbuilder.com). The *phf7* fragments were inserted into their “user-defined promoter” modification of the pUASTattB expression vector. The transgenic constructs were sent to Rainbow Transgenic Flies Inc. for *phi-C31* catalyzed integration into the 65B2 PBac{y[+]-attP-3B}VK00033 site.
(PDF)

S3 Fig The first exon and a portion of the first intron are conserved between *D*. *melanogaster*, *D*. *simulans* and *D*. *yakuba*. Pairwise alignments between the first exon and the adjacent intron were identified using the Basic Local Alignment Search Tool (BLASTn) available at (https://blast.ncbi.nlm.nih.gov).
(PDF)

S4 Fig Reduced IDC-GFP staining in germ cells upon *idc* GLKD. Confocal images of ovaries dissected from control (*nos>white-RNAi*) and mutant (*nos>idc-RNAi*) females carrying the IDC-GFP transgene and stained for GFP (green, white in A’ and B’) and DNA (red). Scale bar 50μm. **(A)** In control ovaries, IDC-GFP staining is observed in both the somatic cells and the germ cells. **(B)** In *idc* GLKD ovaries, no IDC-GFP staining is observed in the germ cells. As expected, staining is still observed in the somatic cells.
(PDF)

S1 Table Primers. (PDF)

S2 Table Buffers for ChIP. (PDF)

S3 Table The published RNA-seq data sets utilized in Fig 4. (PDF)

S4 Table The published RNA-seq data sets utilized in Fig 3. (PDF)

S5 Table Underlying numerical data for graphs in Figs 1, 2, 5 and 7. (XLSX)

## References

[1] Becker JS, Nicetto D, Zaret KS. H3K9me3-Dependent Heterochromatin: Barrier to Cell Fate Changes. Trends in genetics: TIG. 2016;32: 29–41. doi: 10.1016/j.tig.2015.11.001 26675384 PMC4698194

[2] Nicetto D, Zaret KS. Role of H3K9me3 heterochromatin in cell identity establishment and maintenance. Current Opinion in Genetics & Development. 2019;55: 1–10. doi: 10.1016/j.gde.2019.04.013 31103921 PMC6759373

[3] Ninova M, Tóth KF, Aravin AA. The control of gene expression and cell identity by H3K9 trimethylation. Development (Cambridge, England). 2019;146. doi: 10.1242/dev.181180 31540910 PMC6803365

[4] Padeken J, Methot SP, Gasser SM. Establishment of H3K9-methylated heterochromatin and its functions in tissue differentiation and maintenance. Nat Rev Mol Cell Bio. 2022; 1–18. doi: 10.1038/s41580-022-00483-w 35562425 PMC9099300

[5] Martienssen R, Moazed D. RNAi and Heterochromatin Assembly. Csh Perspect Biol. 2015;7: a019323. doi: 10.1101/cshperspect.a019323 26238358 PMC4526745

[6] Zofall M, Yamanaka S, Reyes-Turcu FE, Zhang K, Rubin C, Grewal SIS. RNA elimination machinery targeting meiotic mRNAs promotes facultative heterochromatin formation. Science. 2012;335: 96–100. doi: 10.1126/science.1211651 22144463 PMC6338074

[7] Rechtsteiner A, Costello ME, Egelhofer TA, Garrigues JM, Strome S, Petrella LN. Repression of Germline Genes in Caenorhabditis elegans Somatic Tissues by H3K9 Dimethylation of Their Promoters. Genetics. 2019;212: 125–140. doi: 10.1534/genetics.118.301878 30910798 PMC6499516

[8] Methot SP, Padeken J, Brancati G, Zeller P, Delaney CE, Gaidatzis D, et al. H3K9me selectively blocks transcription factor activity and ensures differentiated tissue integrity. Nat Cell Biol. 2021;23: 1163–1175. doi: 10.1038/s41556-021-00776-w 34737442 PMC8572725

[9] Smolko AE, Shapiro-Kulnane L, Salz HK. The H3K9 methyltransferase SETDB1 maintains female identity in Drosophila germ cells. Nature communications. 2018;9: 4155. doi: 10.1038/s41467-018-06697-x 30297796 PMC6175928

[10] Mochizuki K, Sharif J, Shirane K, Uranishi K, Bogutz AB, Janssen SM, et al. Repression of germline genes by PRC1.6 and SETDB1 in the early embryo precedes DNA methylation-mediated silencing. Nat Commun. 2021;12: 7020. doi: 10.1038/s41467-021-27345-x 34857746 PMC8639735

[11] Karimi MM, Goyal P, Maksakova IA, Bilenky M, Leung D, Tang JX, et al. DNA Methylation and SETDB1/H3K9me3 Regulate Predominantly Distinct Sets of Genes, Retroelements, and Chimeric Transcripts in mESCs. Cell Stem Cell. 2011;8: 676–687. doi: 10.1016/j.stem.2011.04.004 21624812 PMC3857791

[12] Wu K, Liu H, Wang Y, He J, Xu S, Chen Y, et al. SETDB1-Mediated Cell Fate Transition between 2C-Like and Pluripotent States. Cell Reports. 2020;30: 25–36.e6. doi: 10.1016/j.celrep.2019.12.010 31914391

[13] Jiang Y, Loh Y-HE, Rajarajan P, Hirayama T, Liao W, Kassim BS, et al. The methyltransferase SETDB1 regulates a large neuron-specific topological chromatin domain. Nature genetics. 2017;49: 1239–1250. doi: 10.1038/ng.3906 28671686 PMC5560095

[14] Du D, Katsuno Y, Meyer D, Budi EH, Chen S-H, Koeppen H, et al. Smad3-mediated recruitment of the methyltransferase SETDB1/ESET controls Snail1 expression and epithelial-mesenchymal transition. EMBO reports. 2018;19: 135–155. doi: 10.15252/embr.201744250 29233829 PMC5757214

[15] Koide S, Oshima M, Takubo K, Yamazaki S, Nitta E, Saraya A, et al. Setdb1 maintains hematopoietic stem and progenitor cells by restricting the ectopic activation of nonhematopoietic genes. Blood. 2016;128: 638–649. doi: 10.1182/blood-2016-01-694810 27301860

[16] Schultz DC, Ayyanathan K, Negorev D, Maul GG, Rauscher FJ. SETDB1: a novel KAP-1-associated histone H3, lysine 9-specific methyltransferase that contributes to HP1-mediated silencing of euchromatic genes by KRAB zinc-finger proteins. Genes & Development. 2002;16: 919–932. doi: 10.1101/gad.973302 11959841 PMC152359

[17] Tan S-L, Nishi M, Ohtsuka T, Matsui T, Takemoto K, Kamio-Miura A, et al. Essential roles of the histone methyltransferase ESET in the epigenetic control of neural progenitor cells during development. Development (Cambridge, England). 2012;139: 3806–3816. doi: 10.1242/dev.082198 22991445

[18] Cheng E-C, Hsieh C-L, Liu N, Wang J, Zhong M, Chen T, et al. The Essential Function of SETDB1 in Homologous Chromosome Pairing and Synapsis during Meiosis. Cell Reports. 2021;34: 108575. doi: 10.1016/j.celrep.2020.108575 33406415 PMC8513770

[19] Lohmann F, Loureiro J, Su H, Fang Q, Lei H, Lewis T, et al. KMT1E mediated H3K9 methylation is required for the maintenance of embryonic stem cells by repressing trophectoderm differentiation. Stem cells (Dayton, Ohio). 2010;28: 201–212. doi: 10.1002/stem.278 20014010

[20] Bilodeau S, Kagey MH, Frampton GM, Rahl PB, Young RA. SetDB1 contributes to repression of genes encoding developmental regulators and maintenance of ES cell state. Genes & Development. 2009;23: 2484–2489. doi: 10.1101/gad.1837309 19884255 PMC2779743

[21] McCarthy RL, Kaeding KE, Keller SH, Zhong Y, Xu L, Hsieh A, et al. Diverse heterochromatin-associated proteins repress distinct classes of genes and repetitive elements. Nat Cell Biol. 2021;23: 905–914. doi: 10.1038/s41556-021-00725-7 34354237 PMC9248069

[22] Nicetto D, Donahue G, Jain T, Peng T, Sidoli S, Sheng L, et al. H3K9me3-heterochromatin loss at protein-coding genes enables developmental lineage specification. Science. 2019;363: 294–297. doi: 10.1126/science.aau0583 30606806 PMC6664818

[23] Biferali B, Bianconi V, Perez DF, Kronawitter SP, Marullo F, Maggio R, et al. Prdm16-mediated H3K9 methylation controls fibro-adipogenic progenitors identity during skeletal muscle repair. Sci Adv. 2021;7: eabd9371. doi: 10.1126/sciadv.abd9371 34078594 PMC8172132

[24] Marsano RM, Dimitri P. Constitutive Heterochromatin in Eukaryotic Genomes: A Mine of Transposable Elements. Cells. 2022;11: 761. doi: 10.3390/cells11050761 35269383 PMC8909793

[25] Wallrath LL, Rodriguez-Tirado F, Geyer PK. Shining Light on the Dark Side of the Genome. Cells. 2022;11: 330. doi: 10.3390/cells11030330 35159140 PMC8834555

[26] Janssen A, Colmenares SU, Karpen GH. Heterochromatin: Guardian of the Genome. Annual review of cell and developmental biology. 2018;34: 265–288. doi: 10.1146/annurev-cellbio-100617-062653 30044650

[27] Aravin AA. Pachytene piRNAs as beneficial regulators or a defense system gone rogue. Nat Genet. 2020;52: 644–645. doi: 10.1038/s41588-020-0656-8 32601474

[28] Yang P, Wang Y, Macfarlan TS. The Role of KRAB-ZFPs in Transposable Element Repression and Mammalian Evolution. Trends in genetics: TIG. 2017;33: 871–881. doi: 10.1016/j.tig.2017.08.006 28935117 PMC5659910

[29] Ecco G, Imbeault M, Trono D. KRAB zinc finger proteins. Development (Cambridge, England). 2017;144: 2719–2729. doi: 10.1242/dev.132605 28765213 PMC7117961

[30] Bruno M, Mahgoub M, Macfarlan TS. The Arms Race Between KRAB–Zinc Finger Proteins and Endogenous Retroelements and Its Impact on Mammals. Annu Rev Genet. 2019;53: 1–24. doi: 10.1146/annurev-genet-112618-043717 31518518

[31] Senft AD, Macfarlan TS. Transposable elements shape the evolution of mammalian development. Nat Rev Genet. 2021;22: 691–711. doi: 10.1038/s41576-021-00385-1 34354263

[32] Benner L, Castro EA, Whitworth C, Venken KJT, Yang H, Fang J, et al. Drosophila Heterochromatin Stabilization Requires the Zinc-Finger Protein Small Ovary. Genetics. 2019;213: 877–895. doi: 10.1534/genetics.119.302590 31558581 PMC6827387

[33] Jankovics F, Bence M, Sinka R, Faragó A, Bodai L, Pettkó-Szandtner A, et al. Drosophila small ovary gene is required for transposon silencing and heterochromatin organization, and ensures germline stem cell maintenance and differentiation. Development (Cambridge, England). 2018;145: dev170639. doi: 10.1242/dev.170639 30389853

[34] Andreev VI, Yu C, Wang J, Schnabl J, Tirian L, Gehre M, et al. Panoramix SUMOylation on chromatin connects the piRNA pathway to the cellular heterochromatin machinery. Nat Struct Mol Biol. 2022;29: 130–142. doi: 10.1038/s41594-022-00721-x 35173350 PMC11749891

[35] Baumgartner L, Handler D, Platzer SW, Yu C, Duchek P, Brennecke J. The Drosophila ZAD zinc finger protein Kipferl guides Rhino to piRNA clusters. Elife. 2022;11. doi: 10.7554/eLife.80067 36193674 PMC9531945

[36] Smolko AE, Shapiro-Kulnane L, Salz HK. An autoregulatory switch in sex-specific phf7 transcription causes loss of sexual identity and tumors in the Drosophila female germline. Development (Cambridge, England). 2020. doi: 10.1242/dev.192856 32816970 PMC7502600

[37] Yang SY, Baxter EM, Doren M van. Phf7 controls male sex determination in the Drosophila germline. Developmental Cell. 2012;22: 1041–1051. doi: 10.1016/j.devcel.2012.04.013 22595675 PMC3635078

[38] Rogers RL, Shao L, Sanjak JS, Andolfatto P, Thornton KR. Revised Annotations, Sex-Biased Expression, and Lineage-Specific Genes in the Drosophila melanogaster Group. G3 Genes Genomes Genetics. 2014;4: 2345–2351. doi: 10.1534/g3.114.013532 25273863 PMC4267930

[39] VanKuren NW, Vibranovski MD. A Novel Dataset for Identifying Sex-Biased Genes in Drosophila. J Genom. 2014;2: 64–67. doi: 10.7150/jgen.7955 25031657 PMC4091448

[40] Osumi K, Sato K, Murano K, Siomi H, Siomi MC. Essential roles of Windei and nuclear monoubiquitination of Eggless/SETDB1 in transposon silencing. Embo Rep. 2019;20: e48296. doi: 10.15252/embr.201948296 31576653 PMC6893296

[41] Rangan P, Malone CD, Navarro C, Newbold SP, Hayes PS, Sachidanandam R, et al. piRNA production requires heterochromatin formation in Drosophila. Current biology: CB. 2011;21: 1373–1379. doi: 10.1016/j.cub.2011.06.057 21820311 PMC3205116

[42] Sienski G, Dönertas D, Brennecke J. Transcriptional silencing of transposons by Piwi and maelstrom and its impact on chromatin state and gene expression. Cell. 2012;151: 964–980. doi: 10.1016/j.cell.2012.10.040 23159368 PMC3504300

[43] Mohn F, Sienski G, Handler D, Brennecke J. The rhino-deadlock-cutoff complex licenses noncanonical transcription of dual-strand piRNA clusters in Drosophila. Cell. 2014;157: 1364–1379. doi: 10.1016/j.cell.2014.04.031 24906153

[44] Teixeira FK, Okuniewska M, Malone CD, Coux R-X, Rio DC, Lehmann R. piRNA-mediated regulation of transposon alternative splicing in the soma and germ line. Nature. 2017;8: 272. doi: 10.1038/nature25018 29211718 PMC5933846

[45] Fabry MH, Ciabrelli F, Munafò M, Eastwood EL, Kneuss E, Falciatori I, et al. piRNA-guided co-transcriptional silencing coopts nuclear export factors. Elife. 2019;8: e47999. doi: 10.7554/eLife.47999 31219034 PMC6677536

[46] Théron E, Maupetit-Mehouas S, Pouchin P, Baudet L, Brasset E, Vaury C. The interplay between the Argonaute proteins Piwi and Aub within Drosophila germarium is critical for oogenesis, piRNA biogenesis and TE silencing. Nucleic Acids Res. 2018;46: gky695–. doi: 10.1093/nar/gky695 30113668 PMC6212714

[47] Yu Y, Gu J, Jin Y, Luo Y, Preall JB, Ma J, et al. Panoramix enforces piRNA-dependent cotranscriptional silencing. Science. 2015;350: 339–342. doi: 10.1126/science.aab0700 26472911 PMC4722808

[48] Murano K, Iwasaki YW, Ishizu H, Mashiko A, Shibuya A, Kondo S, et al. Nuclear RNA export factor variant initiates piRNA-guided co-transcriptional silencing. Embo J. 2019;38: e102870. doi: 10.15252/embj.2019102870 31368590 PMC6717896

[49] Sienski G, Batki J, Senti K-A, Dönertas D, Tirian L, Meixner K, et al. Silencio/CG9754 connects the Piwi-piRNA complex to the cellular heterochromatin machinery. Genes & Development. 2015;29: 2258–2271. doi: 10.1101/gad.271908.115 26494711 PMC4647559

[50] Batki J, Schnabl J, Wang J, Handler D, Andreev VI, Stieger CE, et al. The nascent RNA binding complex SFiNX licenses piRNA-guided heterochromatin formation. Nat Struct Mol Biol. 2019;26: 720–731. doi: 10.1038/s41594-019-0270-6 31384064 PMC6828549

[51] Zhao K, Cheng S, Miao N, Xu P, Lu X, Zhang Y, et al. A Pandas complex adapted for piRNA-guided transcriptional silencing and heterochromatin formation. Nat Cell Biol. 2019;21: 1261–1272. doi: 10.1038/s41556-019-0396-0 31570835

[52] Chung H-R, Schäfer U, Jäckle H, Böhm S. Genomic expansion and clustering of ZAD-containing C2H2 zinc-finger genes in Drosophila. EMBO reports. 2002;3: 1158–1162. doi: 10.1093/embo-reports/kvf243 12446571 PMC1308319

[53] Chung H-R, Löhr U, Jäckle H. Lineage-specific expansion of the zinc finger associated domain ZAD. Molecular biology and evolution. 2007;24: 1934–1943. doi: 10.1093/molbev/msm121 17569752

[54] Kasinathan B, Colmenares SU, McConnell H, Young JM, Karpen GH, Malik HS. Innovation of heterochromatin functions drives rapid evolution of essential ZAD-ZNF genes in Drosophila. eLife. 2020;9. doi: 10.7554/eLife.63368 33169670 PMC7655104

[55] Shapiro-Kulnane L, Bautista O, Salz HK. An RNA-interference screen in Drosophila to identify ZAD-containing C2H2 zinc finger genes that function in female germ cells. G3 Genes Genomes Genetics. 2020;11: jkaa016. doi: 10.1093/g3journal/jkaa016 33561227 PMC8022714

[56] Jauch R, Bourenkov GP, Chung H-R, Urlaub H, Reidt U, Jäckle H, et al. The zinc finger-associated domain of the Drosophila transcription factor grauzone is a novel zinc-coordinating protein-protein interaction module. Structure (London, England: 1993). 2003;11: 1393–1402. doi: 10.1016/j.str.2003.09.015 14604529

[57] Zolotarev N, Fedotova A, Kyrchanova O, Bonchuk A, Penin AA, Lando AS, et al. Architectural proteins Pita, Zw5,and ZIPIC contain homodimerization domain and support specific long-range interactions in Drosophila. Nucleic Acids Res. 2016;44: 7228–7241. doi: 10.1093/nar/gkw371 27137890 PMC5009728

[58] Maksimenko O, Kyrchanova O, Klimenko N, Zolotarev N, Elizarova A, Bonchuk A, et al. Small Drosophila zinc finger C2H2 protein with an N-terminal zinc finger-associated domain demonstrates the architecture functions. Biochimica Et Biophysica Acta Bba—Gene Regul Mech. 2020;1863: 194446. doi: 10.1016/j.bbagrm.2019.194446 31706027

[59] Bonchuk AN, Boyko KM, Nikolaeva AY, Burtseva AD, Popov VO, Georgiev PG. Structural insights into highly similar spatial organization of zinc-finger associated domains with a very low sequence similarity. Structure. 2022;30: 1004–1015.e4. doi: 10.1016/j.str.2022.04.009 35580610

[60] Bonchuk A, Boyko K, Fedotova A, Nikolaeva A, Lushchekina S, Khrustaleva A, et al. Structural basis of diversity and homodimerization specificity of zinc-finger-associated domains in Drosophila. Nucleic Acids Res. 2021;49: 2375–2389. doi: 10.1093/nar/gkab061 33638995 PMC7913770

[61] Shapiro-Kulnane L, Smolko AE, Salz HK. Maintenance of Drosophila germline stem cell sexual identity in oogenesis and tumorigenesis. Development (Cambridge, England). 2015;142: 1073–1082. doi: 10.1242/dev.116590 25758221 PMC4360176

[62] Brown JB, Boley N, Eisman R, May GE, Stoiber MH, Duff MO, et al. Diversity and dynamics of the Drosophila transcriptome. Nature. 2014;512: 393–399. doi: 10.1038/nature12962 24670639 PMC4152413

[63] Hinnant TD, Merkle JA, Ables ET. Coordinating Proliferation, Polarity, and Cell Fate in the Drosophila Female Germline. Frontiers in cell and developmental biology. 2020;8: 19. doi: 10.3389/fcell.2020.00019 32117961 PMC7010594

[64] Chau J, Kulnane LS, Salz HK. Sex-lethal facilitates the transition from germline stem cell to committed daughter cell in the Drosophila ovary. Genetics. 2009;182: 121–132. doi: 10.1534/genetics.109.100693 19237687 PMC2674811

[65] Chau J, Kulnane LS, Salz HK. Sex-lethal enables germline stem cell differentiation by down-regulating Nanos protein levels during Drosophila oogenesis. Proceedings of the National Academy of Sciences of the United States of America. 2012;109: 9465–9470. doi: 10.1073/pnas.1120473109 22645327 PMC3386055

[66] Li Y, Minor NT, Park JK, Mckearin DM, Maines JZ. Bam and Bgcn antagonize Nanos-dependent germ-line stem cell maintenance. Proceedings of the National Academy of Sciences. 2009;106: 9304–9309. doi: 10.1073/pnas.0901452106 19470484 PMC2695086

[67] Yi X, Vries HI de, Siudeja K, Rana A, Lemstra W, Brunsting JF, et al. Stwl modifies chromatin compaction and is required to maintain DNA integrity in the presence of perturbed DNA replication. Molecular biology of the cell. 2009;20: 983–994. doi: 10.1091/mbc.e08-06-0639 19056684 PMC2633405

[68] Shokri L, Inukai S, Hafner A, Weinand K, Hens K, Vedenko A, et al. A Comprehensive Drosophila melanogaster Transcription Factor Interactome. Cell Reports. 2019;27: 955–970.e7. doi: 10.1016/j.celrep.2019.03.071 30995488 PMC6485956

[69] Zinshteyn D, Barbash DA. Stonewall prevents expression of ectopic genes in the ovary and accumulates at insulator elements in D. melanogaster. Plos Genet. 2022;18: e1010110. doi: 10.1371/journal.pgen.1010110 35324887 PMC8982855

[70] Allshire RC, Madhani HD. Ten principles of heterochromatin formation and function. Nature reviews Molecular cell biology. 2018;19: 229–244. doi: 10.1038/nrm.2017.119 29235574 PMC6822695

[71] Sarkar K, Kotb NM, Lemus A, Martin ET, McCarthy A, Camacho J, et al. A feedback loop between heterochromatin and the nucleopore complex controls germ-cell to oocyte transition during Drosophila oogenesis. Biorxiv. 2021; 2021.10.31.466575. doi: 10.1101/2021.10.31.466575 PMC11301765 37673064

[72] Sarov M, Barz C, Jambor H, Hein MY, Schmied C, Suchold D, et al. A genome-wide resource for the analysis of protein localisation in Drosophila. Elife. 2016;5: e12068. doi: 10.7554/eLife.12068 26896675 PMC4805545

[73] Hu Y, Comjean A, Rodiger J, Liu Y, Gao Y, Chung V, et al. FlyRNAi.org—the database of the Drosophila RNAi screening center and transgenic RNAi project: 2021 update. Nucleic Acids Res. 2020;49: gkaa936–. doi: 10.1093/nar/gkaa936 33104800 PMC7778949

[74] Gratz SJ, Ukken FP, Rubinstein CD, Thiede G, Donohue LK, Cummings AM, et al. Highly specific and efficient CRISPR/Cas9-catalyzed homology-directed repair in Drosophila. Genetics. 2014;196: 961–971. doi: 10.1534/genetics.113.160713 24478335 PMC3982687

[75] Port F, Strein C, Stricker M, Rauscher B, Heigwer F, Zhou J, et al. A large-scale resource for tissue-specific CRISPR mutagenesis in Drosophila. Elife. 2020;9: e53865. doi: 10.7554/eLife.53865 32053108 PMC7062466

[76] Livak KJ, Schmittgen TD. Analysis of relative gene expression data using real-time quantitative PCR and the 2(-Delta Delta C(T)) Method. Methods (San Diego, Calif). 2001;25: 402–408. doi: 10.1006/meth.2001.1262 11846609

