# Supplementary material for: Safeguarding Drosophila female germ cell identity depends on an H3K9me3 mini domain guided by a ZAD zinc finger protein
Source: PLoS Genet. 2022 Dec 22;18(12):e1010568. doi: 10.1371/journal.pgen.1010568 (PMC9822104; doi:10.1371/journal.pgen.1010568)

S1 Fig. The first intron of *phf7* contains seven copies of an ~250 bp DNA sequence
Sequence alignment of the 7 ~250 bp sequences generated with the multiple sequence alignment tool MUSCLE embedded in the SnapGene software (snapgene.com).

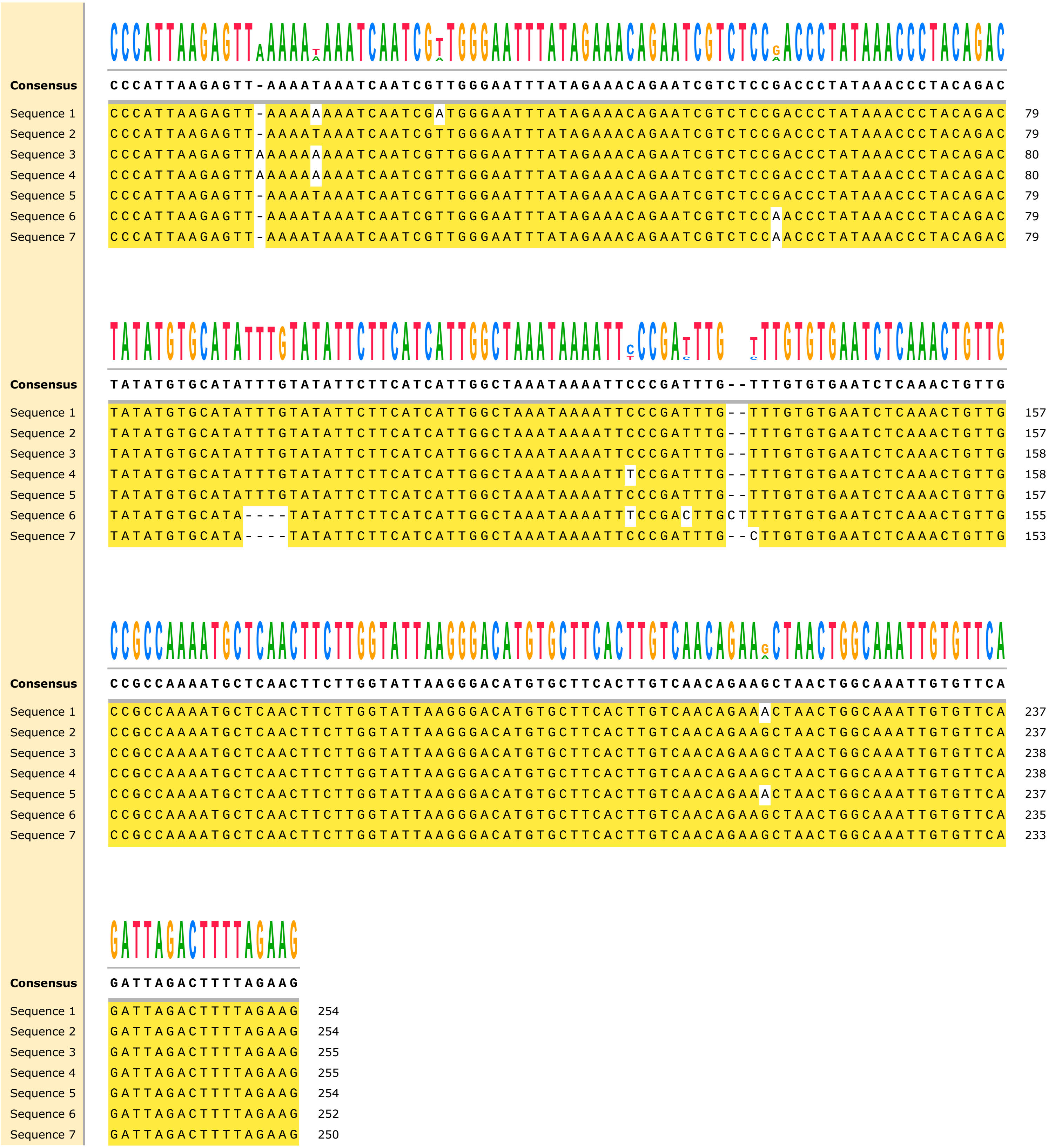

Supplement: S1 Fig — Sequence alignment of the 7 ~250 bp sequences generated with the multiple sequence alignment tool MUSCLE embedded in the SnapGene software (snapgene.com). (PDF) [file pgen.1010568.s001.pdf]
