# Supplementary material for: Safeguarding Drosophila female germ cell identity depends on an H3K9me3 mini domain guided by a ZAD zinc finger protein
Source: PLoS Genet. 2022 Dec 22;18(12):e1010568. doi: 10.1371/journal.pgen.1010568 (PMC9822104; doi:10.1371/journal.pgen.1010568)
Supplement: S2 Fig — ΔR transgene was designed to mimic the 5’ end of the phf7ΔR mutant gene, extending from the first male specific exon to the beginning of the open reading frame in exon 2. In the sequence below, the deleted R element is highlighted in purple. The ΔAR transgene contains a 2nd deletion, highlighted in yellow (region A). The sequences highlighted in blue and the primers located in region B). The constructs were generated by VectorBuilder’s custom cloning services (https://en.vectorbuilder.com). The phf7 fragments were inserted into their “user-defined promoter” modification of the pUASTattB expression vector. The transgenic constructs were sent to Rainbow Transgenic Flies Inc. for phi-C31 catalyzed integration into the 65B2 PBac{y[+]-attP-3B}VK00033 site. (PDF) [file pgen.1010568.s002.pdf]

S2 Figure. **Generation of the ΔR and ΔAR chromatin transgenic reporter lines.** ΔR transgene was designed to mimic the 5' end of the $phf7^{\Delta R}$ mutant gene, extending from the first male specific exon to the beginning of the open reading frame in exon 2. In the sequence below, the deleted R element is highlighted in purple. We note that we left in a single repeat (underlined). The ΔAR transgene contains a 2nd deletion, highlighted in yellow (region A). The sequences highlighted in blue and the primers located in region B). The constructs were generated by VectorBuilder's custom cloning services (https://en.vectorbuilder.com). The *phf7* fragments were inserted into their "user-defined promoter" modification of the pUASTattB expression vector. The transgenic constructs were sent to Rainbow Transgenic Flies Inc. for *phi-C31* catalyzed integration into the 65B2 PBac{y[+]-attP-3B}VK00033 site.

CAGAAGCTCACAGGTCAGAGAAGAAATTTTCGGAATAAAAAAATAAATATAACAAGTTTTCAAAAATAACTC
CGCACCAAACCCATTTAACCAAATCAAAAGCTTAAAGCAGTGAATAGTGTTATTTTTAAAAATTTAGCACAA
ATAAAAAGTTCGGGAATTCAACGCTTTTTGgtaagttttcataatgaaatagcattgtgcaatgaattgtaacacaaatacatatatctactt
gaaaaggcattaattagaacccctatacacatactttgaaaataaaggactgcaactttcattcaattcgcatttgccccaggttacggcaattttcgaggaaat
tcgtagaaacagaaataaaaccgagagtttgacgcttgccaactctggcagtagggttgcccctaagcgtttttccaactgtttaattatttgtgcaggactttac
attgaattggcacacgtgtttttgagacagccacaaaacaaaatcttcaactgaaacccgccatgtttttgttatgctagccgaatgtatataaatcaaagaag
cgtttccgaccccataaaccttacaaactatatatatatatatgttatacatatattcttgatcatttgcttaataatattcccgatttgcttgtgtgaatctcaaactgttg
ccgccaacttcttggtattaagggacacgtgcttcacttgtcaacagaagctaactggcaaattgtgttcagattagactcttagaaacccattaagagttaaaa
aaaatcaatcgatgggaatttatagaaacagaatcgtctccgaccctataaaccctacagactatatgtgcatatttgtatattcttcatcattggctaaataaaa
ttcccgatttgtttgtgtgaatctcaaactgttgccgccaaaatgctcaacttcttggtattaagggacatgtgcttcacttgtcaacagaaactaactggcaaattg
tgttcagattagacttttagaagcccattaagagttaaaataaatcaatcgttgggaatttatagaaacagaatcgtctccgaccctataaaccctacagactat
atgtgcatatttgtatattcttcatcattggctaaataaaattcccgatttgtttgtgtgaatctcaaactgttgccgccaaaatgctcaacttcttggtattaagggac
atgtgcttcacttgtcaacagaagctaactggcaaattgtgttcagattagacttttagaagcccattaagagttaaaaaaaaatcaatcgttgggaatttatag
aaacagaatcgtctccgaccctataaaccctacagactatatgtgcatatttgtatattcttcatcattggctaaataaaattcccgatttgtttgtgtgaatctcaaa
ctgttgccgccaaaatgctcaacttcttggtattaagggacatgtgcttcacttgtcaacagaagctaactggcaaattgtgttcagattagacttttagaagccc
attaagagttaaaaaaaaatcaatcgttgggaatttatagaaacagaatcgtctccgaccctataaaccctacagactatatgtgcatatttgtatattcttcatc
attggctaaataaaatttccgatttgtttgtgtgaatctcaaactgttgccgccaaaatgctcaacttcttggtattaagggacatgtgcttcacttgtcaacagaag
ctaactggcaaattgtgttcagattagacttttagaagcccattaagagttaaaataaatcaatcgttgggaatttatagaaacagaatcgtctccgaccctata
aaccctacagactatatgtgcatatttgtatattcttcatcattggctaaataaaattcccgatttgtttgtgtgaatctcaaactgttgccgccaaaatgctcaacttc
ttggtattaagggacatgtgcttcacttgtcaacagaaactaactggcaaattgtgttcagattagacttttagaagcccattaagagttaaaataaatcaatcgt
tgggaatttatagaaacagaatcgtctccaaccctataaaccctacagactatatgtgcatatatattcttcatcattggctaaataaaatttccgacttgcttttgtg
tgaatctcaaactgttgccgccaaaatgctcaacttcttggtattaagggacatgtgcttcacttgtcaacagaagctaactggcaaattgtgttcagattagact
tttagaagcccattaagagttaaaataaatcaatcgttgggaatttatagaaacagaatcgtctccaaccctataaccctacagactatatgtgcatatatattct
tcatcattggctaaataaaattcccgatttgcttgtgtgaatctcaaactgttgccgccaaaatgctcaacttcttggtattaagggacatgtgcttcacttgtcaac
agaagctaactggcaaattgtgttcagattagacttttagaagcccattaacattcaatcgctgcgcgctctacagcactcgctcttctatttgaatattgccatttc
tctcgcaccacctgagtaacgggtaaataagagctgtttttatttttcttttgaatttcgtttcaatttagctacggcaattctctttggacttcccaaggcaacaaatt
aaaaagatctatttttaatttaagggttgcttaggaaaatataaaactatatatagaaactagaaaagtcaagtattaagattttatatttatatttttctatttaggtt
gttgcttgattttttatttcgttcctatttagctacgtcaattctctttggacttcccaaggcaaaatttgaaaatatccctttttaacatcagggttgcttaagaaaatat
agaaatatatatagaaactagacatgttaaatattaagattttaattaaaatttttcagaacagCAGTTTTAGGCAATTCGTGCCAAGGCAA
ATAAAATCAAATCAACTCGAGGCGATCTGGCATCTCTGGCTGCAGGGCTATCTCCGATAAACCGACGTTC
ATTGCGCGCGAAAACAGTCAATCGCTTCTCAATCGCCTAGATTTGTTTTTTTCGGCTGCTGTTGCTGTTTTA
GTTGAGGGATTGGTCACCGGAAACGCATGCGGCCGCGGACATATGCACACCTGCGATCGTAGTGCCCCA
ACTGGGGTAACCTTTGGGCTCCCCGGGCGCGTACTCCACGAATTC
