# Supplementary material for: Safeguarding Drosophila female germ cell identity depends on an H3K9me3 mini domain guided by a ZAD zinc finger protein
Source: PLoS Genet. 2022 Dec 22;18(12):e1010568. doi: 10.1371/journal.pgen.1010568 (PMC9822104; doi:10.1371/journal.pgen.1010568)
Supplement: S3 Fig — Pairwise alignments between the first exon and the adjacent intron were identified using the Basic Local Alignment Search Tool (BLASTn) available at (https://blast.ncbi.nlm.nih.gov). (PDF) [file pgen.1010568.s003.pdf]

S3 Fig. The first exon and a portion of the first intron are conserved between *D. melanogaster, D. simulans* and *D. yakuba.* Pairwise alignments between the first exon and the adjacent intron were identified using the Basic Local Alignment Search Tool (BLASTn) available at (https://blast.ncbi.nlm.nih.gov).

***D. melanogaster phf7* 1st intron compared to *D. simulans* intron**

```
Score:224 bits(248), Expect:6e-62,
Identities:179/213(84%),  Gaps:7/213(3%), Strand: Plus/Minus

Query  1     GTAAGTTTTCATAATGAAATAGCATTGTGCAATGAATTGTAACACAAATACATATATCTA  60
             ||||||||| ||| |||| || |||||||||| |||||| |||||||||| |||||
Sbjct  1032  GTAAGTTTTTATAGTGAATTACCATTGTGCAAAGAATTGAAACACAAATATATATA----  977

Query  61    CTTGAAAAGGCATTAATTAGAACCCCTATACACATACTTTGAAAATAAAGGACTGCAACT  120
             |||||||||| ||| | || ||||  |||||||| |||||||||| ||||||||||||||
Sbjct  976   CTTGAAAAGGAATTTAATAAAACC--TATACACACACTTTGAAAAAAAAGGACTGCAACT  919

Query  121   TTCATTCAATTCGCATTTGCCCCAGGTTACGGCAATTTTCG-AGGAAATTCGTAGAAACA  179
             | |||||||||| |||||||| |  ||| |  ||||||| | ||| |||||||||||| |
Sbjct  918   TCCATTCAATTCCCATTTGCCGCGAGTTGCACCAATTTTTGAAGGGAATTCGTAGAAATA  859

Query  180   GAAATAAAACCGAGAGTTTGACGCTTGCCAACT  212
             |||||||| ||| | ||||||||||||||||||
Sbjct  858   GAAATAAAGCCGCGCGTTTGACGCTTGCCAACT  826
```

***D. melanogaster phf7* 1st intron compared to *D. yakuba* intron**

```
Score:101 bits(111), Expect:2e-24,
Identities:169/241(70%),  Gaps:24/241(9%), Strand: Plus/Plus

Query  1    GTAAGTTTTCATAATGAAATAGCATTGTGCAATGAATTGTAACACAAATACATATATCTA  60
            |||||||||||||  ||| | ||| |||||||||||||| ||| ||||||        ||
Sbjct  1    GTAAGTTTTCATAGAGAATTGGCAATGTGCAATGAATTGGAACGCAAATA--------TA  52

Query  61   CTTGAAAAGGCATTAATTAGAACCCCTATACACATACTTTGAAAATAAAGGAC--TGCAA  118
             ||| ||||  | ||| ||||  || | |||||| | || |||||  | ||||  | |||
Sbjct  53   TTTGTAAAG--ACTAAATAGA--CCTTTTACACAAATTTAGAAAAGGAGGGACTTTCCAA  108

Query  119  CTTTCATTCAATTCGCATTTGCCCCAGGTTACGGC-AATTTTCGAGGAAATTC---GTAG  174
            ||| | |||       ||||   | ||| | || | ||||| | ||  |||||   | |
Sbjct  109  CTTGCTTTC------TATTTCTTCAAGGCTGCGCCAAATTTCCTAGTGAATTCTAAGAAA  162

Query  175  AAACAGAAATAAAACCGAGAGTTTGACGCTTGCCAACTCTGGCAGTAGGGTTGCCCCTAA  234
            ||| | ||||||||| | | ||||| ||| |  ||||||||||||||||||| || || |
Sbjct  163  AAATACAAATAAAACTGTGCGTTTGGCGCATAGCAACTCTGGCAGTAGGGTTCCCTCTCA  222

Query  235  G  235
            |
Sbjct  223  G  223
```

D. melanogaster phf7 1st exon compared to *D. simulans* 1st exon

```
Score:135 bits(149), Expect:1e-35,
Identities:137/173(79%),  Gaps:8/173(4%), Strand: Plus/Plus

Query  5    AGCTCACAGGTCAGAGAAGAAATTTTCGGAATaaaaaaaTAAA---TATAACAAGTTTTC  61
            ||| ||||| || ||||| |||||||||||||||||||| | |   | ||| ||||||
Sbjct  31   AGCACACAGCTCGGAGAATAAATTTTCGGAATAAAAAAAAATACTTTGTAA-AAGTTTA-  88

Query  62   AAAAATAACTCCGCACCAAACCCATTTAACCAAATCAAAAGCTTAAAGCAGTGAATAGTG  121
            |||||||  ||  |  |||||||||||||| ||| | ||||| |||||||||||||||||
Sbjct  89   AAAAATAGTTCACC--CAAACCCATTTAACAAAAACCAAAGCATAAAGCAGTGAATAGTG  146

Query  122  TTATTTTTAAAAATTTAGCAC-AAATAAAAAGTTCGGGAATTCAACGCTTTTT  173
              |||| | |||||||| ||| ||| || |||||  ||||||| | ||| |||
Sbjct  147  CCATTTATCAAAATTTACCACAAAACAACAAGTTTTGGAATTCCATGCTGTTT  199
```

D. melanogaster phf7 1st exon compared to *D. yakuba* 1st exon

```
Query: None Query ID: lcl|Query_46407 Length: 174
>PREDICTED: Drosophila yakuba PHD finger protein 7 (LOC6525106), transcript variant
X1, mRNA
Sequence ID: XM_002100909.4 Length: 2243
Range 1: 34 to 174

Score:64.4 bits(70), Expect:1e-14,
Identities:103/147(70%),  Gaps:11/147(7%), Strand: Plus/Plus

Query  9    CACAGGTCAGAGAAGAAATTTTCGGAATaaaaaaaTAAA-----TATAACAAGTTTTCAA  63
            ||||| |||| |||| | |     ||| |||||| || |     | | ||||||||| ||
Sbjct  34   CACAGCTCAGTGAAGTACT-----GAAAAAAAAATTATACTTTTTTTACAAGTTTTTAA  88

Query  64   AAATAACTCCGCACCAAACCCATTTAACCAAATCAAAAGCTTAAAGCAGTGAATAGTGTT  123
            || |    ||| || |||   | ||| ||||||||||||| || |||| |||| |||   |
Sbjct  89   AATTTT-TCCACATCAACTCTATTGAACCAAATCAAACGCATAAAACAGTAAATTTTACC  147

Query  124  ATTTTTAAAAATTTAGCACAAATAAAA  150
            || ||| |||||||| |||| | ||||
Sbjct  148  ATATTTCAAAATTTACCACACAAAAAA  174
```
