# Supplementary material for: Safeguarding Drosophila female germ cell identity depends on an H3K9me3 mini domain guided by a ZAD zinc finger protein
Source: PLoS Genet. 2022 Dec 22;18(12):e1010568. doi: 10.1371/journal.pgen.1010568 (PMC9822104; doi:10.1371/journal.pgen.1010568)

S4 Fig. Reduced IDC-GFP staining in germ cells upon *idc* GLKD. Confocal images of ovaries dissected from control (*nos>white-RNAi*) and mutant (*nos>idc-RNAi*) females carrying the IDC-GFP transgene and stained for GFP (green, white in A' and B') and DNA (red). Scale bar 50μm. **(A)** In control ovaries, IDC-GFP staining is observed in both the somatic cells and the germ cells. **(B)** In *idc* GLKD ovaries, no IDC-GFP staining is observed in the germ cells. As expected, staining is still observed in the somatic cells.

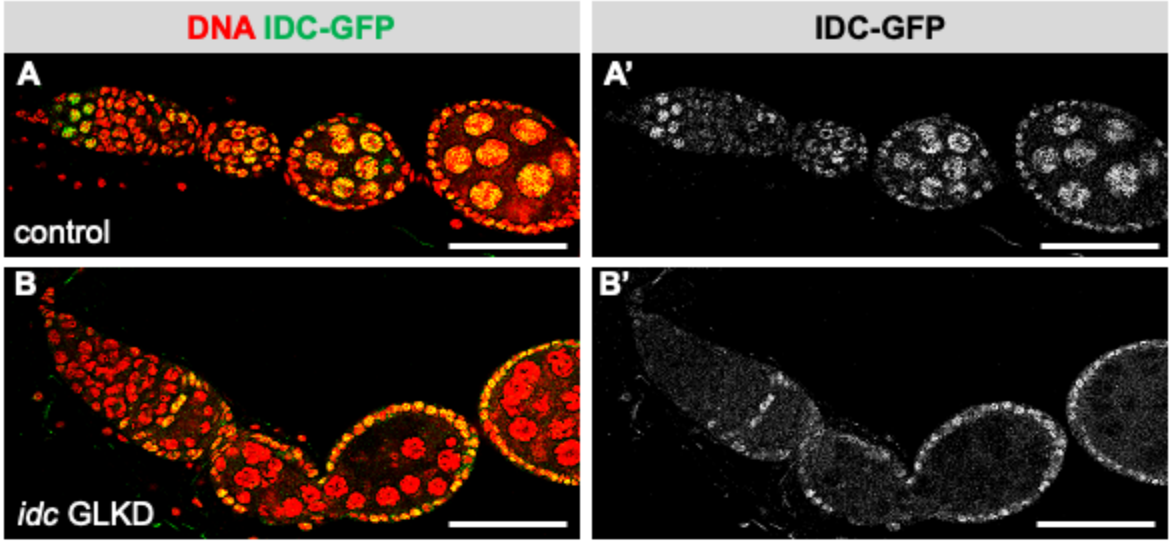

Supplement: S4 Fig — Confocal images of ovaries dissected from control (nos>white-RNAi) and mutant (nos>idc-RNAi) females carrying the IDC-GFP transgene and stained for GFP (green, white in A’ and B’) and DNA (red). Scale bar 50μm. (A) In control ovaries, IDC-GFP staining is observed in both the somatic cells and the germ cells. (B) In idc GLKD ovaries, no IDC-GFP staining is observed in the germ cells. As expected, staining is still observed in the somatic cells. (PDF) [file pgen.1010568.s004.pdf]
