# Supplementary material for: Safeguarding Drosophila female germ cell identity depends on an H3K9me3 mini domain guided by a ZAD zinc finger protein
Source: PLoS Genet. 2022 Dec 22;18(12):e1010568. doi: 10.1371/journal.pgen.1010568 (PMC9822104; doi:10.1371/journal.pgen.1010568)
Supplement: S1 Table — (PDF) [file pgen.1010568.s005.pdf]

S1 Table. Primers

For RT-qPCR experiments

name	forward	reverse
phf7-RC	AGTTCGGGAATTCAACGCTT	GAGATAGCCCTGCAGCCA
total *phf7*	GAGCTGATCTTCGGCACTGT	GCTTCGATGTCCTCCTTGAG
rp49	ATCGGTTACGGATCGAACAA	GACAATCTCCTTGCGCTTCT

For ChIP-qPCR experiments

name	forward	reverse
Intron element A (Fig 1 & 5)	GAGAGTTTGACGCTTGCCAA	CATGGCGGGTTTCAGTTGAA
CDS (Fig 1)	CTGCGACACGTACAACTCGT	AGCGGGCACTTGAAGAAGTA
Rp49 (Fig 1, 2 & 5)	ATCGGTTACGGATCGAACAA	GACAATCTCCTTGCGCTTCT
Intron element B (Fig 2)	AAATTCCCGATTTGCTTGTG	GCGCAGCGATTGAATGTTA
1 (Fig 7)	CAGAAGCTCACAGGTCAGAG	TAAATGGGTTTGGTGCG
2 (Fig 7)	CTCCGCACCAAACCCATTTA	AGCGTTGAATTCCCGAACTT
3 (Fig 7)	GAAATAGCATTGTGCAATGAAT	GCAAATGCGAATTGAATGAAAGT
