# Supplementary material for: Safeguarding Drosophila female germ cell identity depends on an H3K9me3 mini domain guided by a ZAD zinc finger protein
Source: PLoS Genet. 2022 Dec 22;18(12):e1010568. doi: 10.1371/journal.pgen.1010568 (PMC9822104; doi:10.1371/journal.pgen.1010568)
Supplement: S2 Table — (PDF) [file pgen.1010568.s006.pdf]

S2 Table. Buffers for ChIP

Buffer	Components
**PBS (phosphate buffered saline)** pH 7.2	137mM sodium chloride<br>2.7mM potassium chloride<br>10mM sodium phosphate dibasic<br>1.8mM potassium phosphate monobasic
**PBSP**	PBS<br>cOmplete mini EDTA free Protease Inhibitor Cocktail tab (Sigma cat# 11836170001)
**Lysis Buffer 1**	50mM HEPES-KOH (Thermo Fisher cat# 15630080)<br>140mM sodium chloride<br>1mM EDTA (Thermo Fisher cat#AM9261)<br>10% glycerol<br>0.5% Igepal (NP-40)<br>0.25% Triton X-100<br>cOmplete mini EDTA free Protease Inhibitor Cocktail tab (Sigma cat# 11836170001)
**Lysis Buffer 2**	10mM TRIS pH 8 (Sigma cat# T1503)<br>200mM sodium chloride<br>1mM EDTA (Thermo Fisher cat#AM9261)<br>0.5mM EGTA<br>cOmplete mini EDTA free Protease Inhibitor Cocktail tab (Sigma cat# 11836170001)
**Lysis Buffer 3**	10mM TRIS pH 8 (Sigma cat# T1503)<br>100mM sodium chloride<br>1mM EDTA (Thermo Fisher cat#AM9261)<br>0.5mM EGTA<br>0.1% sodium deoxycholate (Sigma cat# D6750)<br>0.5% N-lauroylsarcosine (Sigma cat# L7414)<br>cOmplete mini EDTA free Protease Inhibitor Cocktail tab (Sigma cat# 11836170001)
**ChIP-RIPA**	10mM TRIS pH 8 (Sigma cat# T1503)<br>1mM EDTA (Thermo Fisher cat#AM9261)<br>140mM sodium chloride<br>1% Triton X-100<br>0.1% sodium dodecyl sulfate (Fisher cat# BP-1311-200)<br>0.1% sodium deoxycholate (Sigma cat# D6750)<br>cOmplete mini EDTA free Protease Inhibitor Cocktail tab (Sigma cat# 11836170001)
**ChIP-RIPA/500**	ChIP-RIPA<br>500mM sodium chloride<br>cOmplete mini EDTA free Protease Inhibitor Cocktail tab (Sigma cat# 11836170001)
**ChIP-LiCl**	10mM TRIS pH 8 (Sigma cat# T1503)<br>1mM EDTA (Thermo Fisher cat#AM9261)<br>250mM Lithium Chloride<br>0.5% Igepal (NP-40)<br>0.5% sodium deoxycholate (Sigma cat# D6750)<br>cOmplete mini EDTA free Protease Inhibitor Cocktail tab (Sigma cat# 11836170001)
**TE**	10mM TRIS pH 8 (Sigma cat# T1503)<br>1mM EDTA (Thermo Fisher cat#AM9261)

**Elution Buffer**	10mM TRIS pH 8 (Sigma cat# T1503)<br>5mM EDTA (Thermo Fisher cat#AM9261)<br>300mM sodium chloride<br>0.1% sodium dodecyl sulfate (Fisher cat# BP-1311-200)
