# Supplementary material for: Safeguarding Drosophila female germ cell identity depends on an H3K9me3 mini domain guided by a ZAD zinc finger protein
Source: PLoS Genet. 2022 Dec 22;18(12):e1010568. doi: 10.1371/journal.pgen.1010568 (PMC9822104; doi:10.1371/journal.pgen.1010568)
Supplement: S3 Table — (PDF) [file pgen.1010568.s007.pdf]

S3 Table. The published RNA-seq data sets utilized in Figure 4

Genotype	SRA Run ID	Reference
wild-type (y^1, w^1)	SRR6742503	Smolko et al., 2018 [1]
setdb1 germline RNAi knockdown	GSM2971276	Smolko et al., 2018 [1]
rhino germline RNAi knockdown	GSM3285257	Mohn et. al., 2014 [2]
aub^{HN2}/aub^{QC42}	GSM3284029	Teixeira et al., 2017 [3]
$piwi^1$/$piwi^2$	GSM3284031	Teixeira et al., 2017 [3]
$nxf2^{delta1}$/$nxf1^{F10}$	GSM3443092	Fabry et al., 2019 [4]
$panx^{M1}$/$panx^{M4}$	GSM3443094	Fabry et al., 2019 [4]

1. Smolko AE, Shapiro-Kulnane L, Salz HK. The H3K9 methyltransferase SETDB1 maintains female identity in Drosophila germ cells. Nature communications. 2018;9: 4155. doi:10.1038/s41467-018-06697-x

2. Mohn F, Sienski G, Handler D, Brennecke J. The rhino-deadlock-cutoff complex licenses noncanonical transcription of dual-strand piRNA clusters in Drosophila. Cell. 2014;157: 1364–1379. doi:10.1016/j.cell.2014.04.031

3. Teixeira FK, Okuniewska M, Malone CD, Coux R-X, Rio DC, Lehmann R. piRNA-mediated regulation of transposon alternative splicing in the soma and germ line. Nature. 2017;8: 272. doi:10.1038/nature25018

4. Fabry MH, Ciabrelli F, Munafò M, Eastwood EL, Kneuss E, Falciatori I, et al. piRNA-guided co-transcriptional silencing coopts nuclear export factors. Elife. 2019;8: e47999. doi:10.7554/elife.47999
