# Supplementary material for: Safeguarding Drosophila female germ cell identity depends on an H3K9me3 mini domain guided by a ZAD zinc finger protein
Source: PLoS Genet. 2022 Dec 22;18(12):e1010568. doi: 10.1371/journal.pgen.1010568 (PMC9822104; doi:10.1371/journal.pgen.1010568)
Supplement: S4 Table — (PDF) [file pgen.1010568.s008.pdf]

S4 Table. The published RNA-seq data sets utilized in Figure 3

Species	Tissue	SRA Run ID	Reference Genome	Reference
D. melanogaster	ovary	SRR6742503	dm6	Smolko et al., (2018) [1]
D. melanogaster	testis	SRR070422	dm6	http://modeencode.org
D. simulans	ovary	SRR1511609	ASM175419v2 (GCF_000754195.2)	Rogers et al., (2014) [2]
D. simulans	testis	SRR1520537	ASM175419v2 (GCF_000754195.2)	Rogers et al., (2014) [2]
D. yakuba	ovary	SRR1024009	dyak_caf1 (GCF_000005975.2)	VanKuren and Vibranovski (2014) [3]
D. yakuba	testis	SRR1024006	dyak_caf1 (GCF_000005975.2)	VanKuren and Vibranovski (2014) [3]

1. Smolko AE, Shapiro-Kulnane L, Salz HK. The H3K9 methyltransferase SETDB1 maintains female identity in Drosophila germ cells. Nature communications. 2018;9: 4155. doi:10.1038/s41467-018-06697-x

2. Rogers RL, Shao L, Sanjak JS, Andolfatto P, Thornton KR. Revised Annotations, Sex-Biased Expression, and Lineage-Specific Genes in the Drosophila melanogaster Group. G3 Genes Genomes Genetics. 2014;4: 2345–2351. doi:10.1534/g3.114.013532

3. VanKuren NW, Vibranovski MD. A Novel Dataset for Identifying Sex-Biased Genes in Drosophila. J Genom. 2014;2: 64–67. doi:10.7150/jgen.7955
