# Supplementary material for: Safeguarding Drosophila female germ cell identity depends on an H3K9me3 mini domain guided by a ZAD zinc finger protein
Source: PLoS Genet. 2022 Dec 22;18(12):e1010568. doi: 10.1371/journal.pgen.1010568 (PMC9822104; doi:10.1371/journal.pgen.1010568)
Supplement: Attachment — Submitted filename: response.pdf [file pgen.1010568.s010.pdf]

We thank the three reviewers for investing the time necessary to provide detailed and thoughtful suggestions. We have made every attempt to fully address all comments and believe these revisions have significantly strengthened our manuscript. In response to the reviewer's collective input, we have made the following major changes:

--added a new supplemental figure (Fig S4) to demonstrate RNAi efficiency by showing that *idc GLKD* abolishes the IDC-GFP staining in germ cells but not in the surrounding somatic cells.

--modified the text, modified Fig 2 and inserted a new supplemental figure (Fig S2) to better explain how we generated the ∆R and ∆AR chromatin transgenic reporter lines.

--modified Fig 3B to clarify which non-coding regions are conserved between *D. melanogaster, D. simulans* and *D. yakuba.*

--clarified the section describing the IDC expression pattern in the ovary and other tissues.

--made changes to the text to clarify where IDC binds and address the question of sufficiency as commented in point #5 by reviewer 2.

--modified the model in Fig 7B.

Our response to the reviewers, in **red**, are given in a point-by-point manner below. We have uploaded a revised version of the manuscript in which the major changes are highlighted in yellow.

Reviewer #1

This is a well-written paper with a series of logical, well executed experiments that support the author's conclusion that the ZAD zinc-finger protein IDC plays a direct role in establishing the H3K9me3 domain that regulates the expression of the phf7 gene in the ovary. The importance of this finding is well described in the manuscript: while a lot is known about how H3K27me3-repressive chromatin domains are established, little is known about how small H3K9me3 domains are established. Making good use of public data, the authors clearly show that the piRNA pathway, important in forming large H3K9me3 domains, is not required for the formation of the small H3K9me3 domain that covers the male-specific phf7 promoter. The authors identified the ZAD zinc-finger gene IDC in a previous RNAi screen as a candidate for a phf7 transcriptional regulator. Here they show that (1) knocking down idc with RNAi in ovaries leads to the production of the male-specific phf7 RNA and production of the phf7 protein in ovaries 2) H3K9me3 levels are reduced over the phf7 gene in idc germline mutant clones and (3) IDC binds to the phf7 gene. This paper is made even more interesting by the potential similarities between the recruitment of the H3K9me3 methyltransferase by the KRAB-zinc finger family in mammals and the ZAD zinc-finger family in flies.

Thank you.

I have a few questions:

1) Regarding Fig. 1A and the discussion of the repetitive element: Does the phf7 gene from the related Drosophila species also have a repetitive element (perhaps unrelated)?

Interestingly, we did not find a repetitive element in the introns of either *D. similans* or *D. yakuba*.

2) Regarding Fig. 2A. It's not clear to me exactly what is in the transgene. From the diagram it looks like TSS1 and part of the non-coding region upstream of TSS2 are in the transgene, however, the title of the figure is "Non-coding sequences within the first intron are sufficient for H3K9me3 deposition." This is important because IDC binds to TSS1. If the exons are included then change the title to "Non-coding sequences within the first intron are required for H3K9me3 deposition"

Sorry for the confusion. The first exon is included in the transgenes. To clarify, we have modified Fig 2. We have added details about the construction in Fig S2.

We have also changed the title of Fig 2 to read: *"Non-coding sequences within the first intron are required for H3K9me3 deposition."*

And modified the text to read:

*(Lines 133-137) To complement these studies, we used a transgenic approach to identify the sequences capable of promoting H3K9me3 when inserted into a heterologous genomic location on the 3rd chromosome using site specific integration. We first created a transgene, that includes a portion of the phf7$^{\Delta R}$ mutant allele, extending from the first male specific exon to the beginning of the open reading frame in exon 2 (**S2 Fig**).*

3) Fig. 3A, please put a diagram of the vector used to do this experiment. Where were the fragments cloned in? I can't really visualize it based on the description in the methods.

I assume you mean Fig 2A here. As mentioned above we have modified Fig. 2A and added Fig S2 to better explain what is included in the ∆R and ∆AR chromatin transgenic reporter lines.

4) Fig. 3B would be more informative if the rest of the gene was also shown (same as Fig. 1A). It says in the text that the rest of the DNA is not conserved—show this in the figure. Also, please indicate how many bases are conserved, 84% in a region of how many nucleotides, etc. I see the sequence conservation in Fig. S2, just state the number of bases figure 3A and perhaps state that the sequence conservation is in Fig. S2 in the figure legend.

Done. See revised Fig. 3B. As suggested, we added a line in the Figure legend to say that the sequence conservation is in Figure S2.

5) Pg. 11, line 258. "our studies provide one of the first examples of a ZAD-ZNF protein guiding H3K9me-mediated gene silencing." What are the other examples? Please reference.

We can't come up with a reference; as far as we know, our work provides the first example… we were just being cautious in saying one of the first examples. We have therefore changed the text to read the first example (line 265).

Reviewer #2

In this manuscript, Shapiro-Kulnane et al. take advantage of the phf7 locus to investigate how H3K9me3 mediated silencing is promoted at protein coding genes. This builds on prior work from the lab that had identified H3K9me3 deposition over a testis-specific transcription start site for phf7 as important for silencing expression of the male-specific protein. In a recently published screen, the authors identified CG4936 (IDC) as a putative regulator of phf7 expression. Here, they link these two studies by directly showing that IDC binds near the male-specific TSS of phf7 and is essential for H3K9me3. Overall, the manuscript is clear, and the experiments are rigorously performed. However, the impact beyond what has been previously shown by the lab is somewhat limited and additional experiments could strengthen the conclusions and the model.

Thank you. We note that our previously published screen simply identified CG4936 as one of the 8 ZAD-ZNF genes with functions in the female germline, not as a putative *phf7* regulator. In addition to demonstrating that CG4936, now called *idc,* is required for H3K9me3-mediated silencing of *phf7,* we identify required cis-acting sequences, and demonstrate that the piRNA pathway is not involved in silencing. We feel that this work represents a major advance in our understanding of how silencing occurs.

We have modified the text to make it clear that our recently published screen simply identified CG4936 as a gene required for oogenesis, not as a putative regulator of *phf7* expression.

(Lines 188-191) We recently tested the function of 68 of the 93 ZAD-ZNF encoding genes in female germ cells by performing an RNAi screen [55]. This screen identified eight ZAD-ZNF genes required for oogenesis. Here, we focus on CG4936 which we name identity crisis (idc).

Major issues:

1. The major novel finding in this work is the role of IDC in transcriptional repression in the germline, which was already hinted at by the results of the RNAi screen. To further support this conclusion, the authors should validate the specificity of their RNAi construct and/or use an orthogonal approach to confirm the specificity.

We apologize for this omission. We now present confocal images to show that the TRiP short hairpin line we used produces an effective protein knockdown in Figure S4 and modified the body of the text to read:

*(Lines 202-203) We demonstrated knockdown efficiency by showing that idc GLKD significantly reduces IDC protein levels in female germ cells, but not in the surrounding somatic cells (**S4 Fig**).*

The *idc* loss of function is lethal, therefore our analysis of *idc* function in the adult germline necessitates the use of RNAi reagents. We cannot validate our findings by using a different line because there is only one germline optimized shRNA line in existence. We also note that UP-TORR indicates that there are no predicted off-target effects (see screen shot below for HMC05569). We have added a note to the methods section that reads:

(Lines 323-324). Although off-target effects remain a concern for RNAi-induced knockdown studies, neither RNAi line is predicted to have off-target activity (https://www.flyrnai.org/up-torr/UptorrFly.jsp).

UP-TORR Updated Targets of RNAi Reagents

Download Excel

Search Term	Reagent ID	Type	Vector	Seq Len	Target	No. Genes	OTE (15bp)	CAR CAN	Gene ID(s)	Gene Symbol(s)	Target Type	Isoforms	Target information					
													Name	Len	CDS	Align	Region	Match
CG4936	BKN25003	DKFZ dsRNAs		537	Target 1 gene, all isoform(s)	1	2	0/0	FBgn0038768	CG4936	mRNA	1 of 1	CG4936-RA	1824	118 1683	359 895	CDS	537bp perfect match
CG4936	101256	VDRC KK		537	Target 1 gene, all isoform(s)	1	2	0/0	FBgn0038768	CG4936	mRNA	1 of 1	CG4936-RA	1824	118 1683	359 895	CDS	537bp perfect match
CG4936	DRSC15680	DRSC dsRNAs		513	Target 1 gene, all isoform(s)	1	2	0/0	FBgn0038768	CG4936	mRNA	1 of 1	CG4936-RA	1824	118 1683	646 1158	CDS	513bp perfect match
CG4936	DRSC37483	DRSC dsRNAs		350	Target 1 gene, all isoform(s)	1	0	0/0	FBgn0038768	CG4936	mRNA	1 of 1	CG4936-RA	1824	118 1683	308 657	CDS	350bp perfect match
CG4936	6253	VDRC GD		296	Target 1 gene, all isoform(s)	1	1	0/0	FBgn0038768	CG4936	mRNA	1 of 1	CG4936-RA	1824	118 1683	389 684	CDS	296bp perfect match
CG4936	6252	VDRC GD		296	Target 1 gene, all isoform(s)	1	1	0/0	FBgn0038768	CG4936	mRNA	1 of 1	CG4936-RA	1824	118 1683	389 684	CDS	296bp perfect match
CG4936	HMC05569	TRiP Short Hairpin	VALIUM20	21	Target 1 gene, all isoform(s)	1	0	0/0	FBgn0038768	CG4936	mRNA	1 of 1	CG4936-RA	1824	118 1683	309 329	CDS	21bp perfect match

2. The expression pattern of IDC as a whole is not clearly explained. Is IDC expressed broadly in all tissues or only in the germline?

We apologize for not providing enough information about the expression pattern. To make clear that it is likely that IDC is ubiquitously expressed, but that we have limited our analysis to its expression pattern in the ovary, we begin this section with the following statement:

(Line 220-221) Published modENCODE RNA-seq data sets indicates that the idc RNA is broadly expressed throughout development [62]. To examine IDC protein expression in the ovary,…

And modified the description to read:

*(Lines 226-231) We observed IDC-GFP in the somatic cells, the nurse cells and the oocyte (**Fig 6A**). Notably, the_IDC-GFP protein is nuclear. Furthermore, IDC-GFP is tightly associated with the nurse cell polytene chromosomes, consistent with its presumed DNA binding activity (**Fig 6B**).*

*In the germarium, at the anterior end of the ovariole, we observed prominent IDC-GFP staining in the nucleus of only 3 to 6 germ cells (**Fig 6A)**.*

And we end with:

(Lines 242-243) Together, these observations indicate that the nuclear IDC protein is broadly expressed, with prominent expression in the GSCs and their immediate daughter cells.

In the Discussion, the authors mention that IDC is also expressed in male germ cells, suggesting that the simple model presented in Figure 7B is misleading.

This comment led us to redraw the model in Fig 7B.

The authors have all the tools in hand to test if IDC binds phf7 in the male germline. The authors discuss a potential candidate for mediating the female-specific functions (STWL). It is unclear why they do not directly test this hypothesis.

These are interesting and important questions, but they are beyond the scope of our current work.

3. To better understand the function of IDC in the female-specific silencing, it would be useful to determine IDC binding beyond the phf7 locus both in the male and female germlines. This would provide insights into additional targets and possibly more generally into the role of the ZAD-ZNF gene family.

We agree that a better understanding of IDC function will require a global analysis. However, we feel that this line of investigation is well beyond the scope of this work which is focused on *phf7* silencing.

Along these same lines, in Figure 6 IDC-GFP appears to be broadly expressed in the female germline. It would be useful for the authors to test and/or discuss in which cell types IDC is required.

We generated an *idc* null allele and found that the animals do not survive to adulthood (see materials and methods). As our focus is on how *phf7* is silenced in the female germline, we did not look beyond its requirement in the female germline.

Does overexpression of IDC lead to H3K9me3 deposition over phf7?

IDC is broadly expressed, so I am not sure what the impact of overexpression would be.

4. A major conclusion from the manuscript is that the determinants required for H3K9me3 deposition at phf7 are within element A of the 1st intron. However, additional experiments would strengthen this conclusion, which is currently based on the fact that deletion of a set of repeats does not disrupt H3K9me3 while a deletion that also includes the A region does. With the data presented, it remains possible that these two regions are redundant. Deletions of the A region alone should be tested. Likewise, it would be useful to test sufficiency. Does insertion of the A region in a transgene lead to H3K9me3 in the female germline?

These are excellent points. We have modified our conclusions to read:

*(Lines 141-150). As expected, we found that these sequences included in the ΔR transgene promotes H3K9me3 accumulation. (**Fig 2A and Fig 2B**). These data reinforce our conclusion that the tandem repeats (element R) are not required for H3K9me3 accumulation. In contrast, we found that H3K9me3 did not accumulate on a second transgene in which both elements A and R were removed (**Fig 2A and Fig 2B**). We therefore conclude that the sequences that remain, including the first exon and region B, are not sufficient for H3K9me3 recruitment. These results also establish that element A contains cis-regulatory determinants required for H3K9me3 recruitment. It remains to be determined whether the element A determinants are sufficient for H3K9me3 recruitment or function redundantly with sequences within element R.*
.

5. As written, some of the data seems contradictory. The A region in the first intron appears important for H3K9me3 deposition, but IDC appears to bind the exon (Figure 7A). Do the authors think that there are additional factors that recognize the A region? At the very least, the authors should discuss these conflicting data.

We elaborate on this excellent point in the discussion.

(Lines 267-275) Although our work is consistent with a simple model in which the SETDB1 H3K9me3 methyltransferase is recruited to phf7 by IDC, the mechanism by which IDC guides the methylation machinery to phf7 remains an open question. For example, it remains unclear whether recruitment is direct, as our attempts to co-immunoprecipitate SETDB1 and IDC were unsuccessful. Furthermore, while we establish that IDC is required for H3K9me3 recruitment, our chromatin transgenic reporter assays show that the region to which it binds, the conserved first exon, is not sufficient. This observation, together with our identification of a second conserved cis-regulatory element within the adjoining intron invites speculation that IDC works in conjunction with other sequence-specific recruitment factors.

Minor issues:

In line 125 it says the p value is 0.0003, however in the corresponding figure it says the p value is 0.003

Thank you for catching this error. Fixed.

All of the comparisons made with RT-qPCR and ChIP-qPCR should have statistics.

Done

Figure 4 should include scales for the y-axes for the genome browser tracks.

Fixed.

Reviewer #3
Heterochromatin is guided to transposable elements (TEs) to silence their transcription. Sequence-specific transcription factors such as KRAB-ZFPs in mammals and ZAD-ZNF in the fly have been shown to guide this heterochromatin formation. However, how protein-coding genes are targeted for silencing is not fully understood. During, Drosophila oogenesis testis-specific version of PHD finger protein 7 (phf7) transcription is silenced. Using lineage-specific transcription of ph7 as a paradigm, the authors find that member of the ZAD-ZNF protein family that they name Identity Crisis (IDC) is necessary for H3K9me3 deposition in a sequence-specific manner on phf7 gene locus. In this study, the authors specifically claim that:
1. Conserved sequences in the first intron are required for H3K9me3 deposition
2. IDC is expressed in the undifferentiated cells of the germline.
3. IDC silences testis-specific ph7 expression through H3K9me3 deposition dependent on SETDB1 but independent of piRNA machinery.
4. IDC binds to phf7 locus to directly promote silencing of phf7 locus

Major Comments:
1. Figure 1 is superfluous with Figure 2 and can be combined. It was confusing to read that something is not required for first.

While the results reported in Fig. 1 were surprisingly negative, they were our first hint that the H3K9me3 guiding mechanism was novel. Fig. 1, reports on a deletion in the endogenous locus, whereas Fig. 2 reports on H3K9me3 on a fragment of *phf7* inserted on the 3rd chromosome. Because of these different experimental approaches, we think it best that we do not merge Fig. 1 and Fig. 2.

2. IDC RNAi data needs additional validation. The authors have only used one RNAi line. They need to test another RNAi line or mutant clones.

We have validated our RNAi reagent. Please see response to reviewer 2, comment #1.

3. The phenotype of IDC depletion should be described in more detail and compared to SETDB1 mutants.

This is a good point that has led us to more carefully describe what we know about the mutant phenotype. The text now reads:

(Lines 195-197). Interestingly, the knockdown phenotype of IDC suggests a defect prior to oocyte specification [55]. Because this phenotype is reminiscent of the germ cell defects caused by ectopic PHF7 expression, we hypothesize that IDC is required for phf7 repression.

We, and others, have shown that loss of *setdb1* also blocks differentiation (Smolko et al., 2018; Clough et al., 2007; Clough et al., 2014; Rangan et al., 2011; Wang et al., 2011; Yan et al., 2014). However, given that SETDB1 silences other genes, we believe that the more appropriate comparison is to the gain of PHF7 expression.

4. Does the loss of IDC lead to depletion of H3K9me3 at a global level?

This is an interesting and important question. However, we feel that a global analysis is beyond the scope of this analysis as our focus is on *phf7* silencing.

Minor Comments:
1. Figure 3B- the lines in the table are not perpendicular.

Fixed.

2. Y axis for Figure 4 is needed (X TPMs)

Done

3. Figure 5A requires significance

Done.

4. Authors should add grayscale for staining where possible

Done; see Fig. 5, 6 and Fig. S4

5. IDC-GFP quantification of levels as a function of development in the germarium is needed.

We agree that the level of IDC-GFP protein appears to change as germ cells develop. In the germarium, the staining is most prominent in the GSC and their immediate daughter cells. However, we respectively disagree that quantification of the levels as a function of development in the germarium is needed for this publication. The important point is that IDC-GFP is co-expressed in the same cells as SXL, which is indirectly required to maintain *phf7* silencing.
